# Extracellular vesicles derived from the choroid plexus trigger the differentiation of neural stem cells

**DOI:** 10.1002/jev2.12276

**Published:** 2022-11-02

**Authors:** Zuzana Ditte, Ivan Silbern, Peter Ditte, Henning Urlaub, Gregor Eichele

**Affiliations:** ^1^ Department of Genes and Behavior Max Planck Institute for Multidisciplinary Sciences Göttingen Germany; ^2^ Biological Rhythms Max Planck Institute for Dynamics and Self Organization Göttingen Germany; ^3^ The Bioanalytical Mass Spectrometry Group Max Planck Institute for Multidisciplinary Sciences Göttingen Germany; ^4^ Institute for Clinical Chemistry University Medical Center Göttingen Göttingen Germany

**Keywords:** brain ventricles, cerebrospinal fluid, choroid plexus derived vesicle, exosome assay, extracellular vesicles, mass spectrometry, neural stem cell differentiation

## Abstract

The choroid plexus secrets cerebrospinal fluid (CSF) composed of electrolytes, cytokines, growth factors, metabolites and extracellular vesicles (EVs) that flow through the interconnected brain ventricles. On their course, CSF components can act as signals that affect, for example, neural stem cells (NSCs) residing in niches of the ventricular wall. We studied EV‐born CSF signals in an in vitro culture system. We purified EVs from the secretome of a choroid plexus cell line (Z310 cells), and from primary choroid plexus cultures and co‐cultured those EVs with NSCs isolated from the niche of the lateral and the third ventricle. EVs^Z310^ and EVs^CHP^ were purified by differential centrifugation. This yielded fractions of EVs of 50–150‐nm diameter that induced a complex multicellular network formation and NSC differentiation. Both types of EV converted the round NSCs to cells that extended long processes that contacted nearby, alike‐shaped cells. Mass spectrometry showed that the differentiation‐inducing EV^Z310^ were enriched for membrane and membrane‐associated proteins involved in cell differentiation, membrane trafficking, and membrane organization. We hypothesize that this type of EV ^Z310^ cargo causes changes of stem cell morphology that leads to multicellular networks in the niches. This cell‐shape transition may represent an initial step in NSC differentiation.

## 1 INTRODUCTION

**FIGURE 1:**
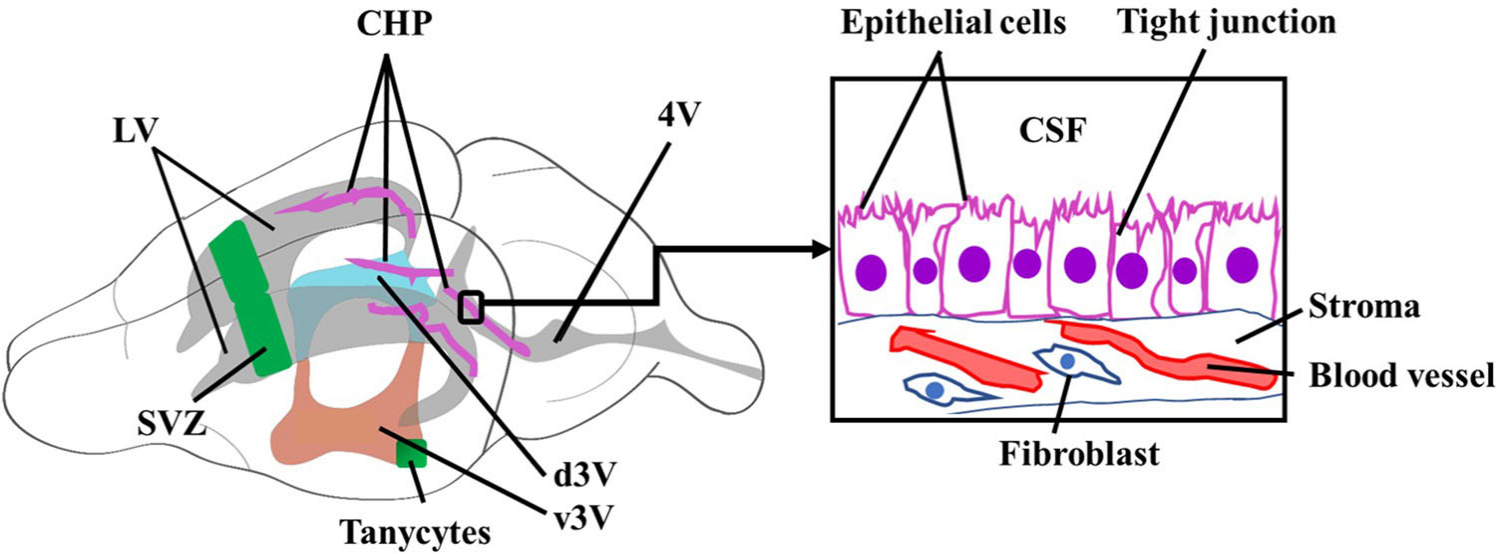
Architecture of the ventricles of the mouse brain. Lateral view of the adult mouse brain showing the ventricular system that consists of two lateral ventricles (LV, grey), the bipartite third ventricle (dorsal 3V, blue [d3V] and ventral 3V, orange [v3V]) and the fourth ventricle (4V, grey). Each ventricle has a choroid plexus (pink) that produces CSF. The choroid plexus (CHP, right box) consists of a secretory polarized epithelial cell layer and stroma composed of fibroblasts (Dorrier et al., 2022), immune cells and perivascular cells. The tight junctions between epithelial cells form the blood–CSF barrier (Ghersi‐Egea et al., 2018). Neurogenic niches are the sub‐ventricular zone (SVZ) and the tanycyte region (both green).

The mammalian ventricular system of the brain consists of two lateral ventricles, the third and the fourth ventricle (Figure 1). The ventricles are filled with cerebrospinal fluid (CSF) which is produced in and flows through the ventricles before being absorbed by arachnoid granulations and the lymphatic system (Hladky & Barrand, 2014). Ependymal cells form the ventricular walls and carry each a bundle of motile cilia that mediates directional CSF flow (Mirzadeh et al., 2010). This directional flow follows precise trajectories (Faubel et al., 2016) suggesting that in this way, CSF components are delivered to specific locations within the ventricular system (Eichele et al., 2020). Most of the CSF is generated and secreted by the choroid plexus (Damkier et al., 2010). CSF protects the brain from mechanical impacts and collects waste from the brain (Cserr, 1971). In addition, CSF contains signalling factors, ions, lipids and hormones required for proper brain development and function (Chau et al., 2015; Kaiser et al., 2019; Lehtinen et al., 2011; Lun et al., 2015; Silva‐Vargas et al., 2016; Zappaterra & Lehtinen, 2012). CSF also contains extracellular vesicles (EVs) (Balusu et al., 2016; Fame & Lehtinen, 2020; Feliciano et al., 2014; Lun et al., 2015; Murillo et al., 2019; Street et al., 2012; Tietje et al., 2014; Vella et al., 2008; Yagi et al., 2017) that are bilayer membrane‐enclosed vesicles, produced and secreted by many cell types. EVs are loaded with proteins and nucleic acids (Johnstone et al., 1987; Kalra et al., 2012; Keerthikumar et al., 2016; Valadi et al., 2007; Van Niel et al., 2006). EVs termed microvesicles (size range of 100–1000 nm) form by outward budding of the plasma membrane. EVs termed exosomes (size 50–150 nm) form by an intracellular endocytic trafficking pathway involving multi‐vesicular endosomes that release exosomes upon fusion with the plasma membrane (Van Niel et al., 2018; Mathieu et al., 2019). Because current purification procedures do not exclude the presence of microvesicles in exosome preparations and vice versa, one refers to these vesicles collectively as ‘EVs’.

EVs mediate communication in vitro and in vivo (Mathieu et al., 2019; Van Niel et al., 2018) between different cell types such as neurons and oligodendrocytes (Frühbeis et al., 2013), neurons and astroglia (Men et al., 2019; Morel et al., 2013) and neurons and Schwann cells (Lopez‐Verrilli et al., 2013). EVs involved in intercellular communication do so in the pre‐metastatic niche (Shurtleff et al., 2018), for instance by promoting breast cancer cells motility via Wnt‐planar cell polarity signalling (Luga et al., 2012). EVs also play a role in the mesenchymal stem cell niche (Hayashi et al., 2017) and in the epithelial–mesenchymal niche (Nakano et al., 2017). Glioma‐derived EVs drive the differentiation of embryonic neural stem cells (NSCs) to astrocytes (Sharma et al., 2020) and EVs are thought to regulate neurogenesis in the early postnatal mouse brain (Sharma et al., 2019). Recently, it was shown that Cyclin D1 contained in EVs produced by PC12 and N2A cell lines promote neural differentiation of embryonic stem cells (Song et al., 2021).

Injecting fluorescently labelled choroid plexus‐derived EVs into the lateral ventricle showed that EVs could cross the ependymal cell layer and reach brain parenchyma (Balusu et al., 2016; Grapp et al., 2013). EVs have various effects on the nervous system. Balusu et al. (2016) showed that systemic inflammation of mice by intraperitoneal injection of lipopolysaccharide or tumour necrosis factor induced an increase in pro‐inflammatory miRNAs in EVs produced by choroid plexus. These EVs also interacted with astrocytes and microglia to up‐regulate inflammatory genes (Balusu et al., 2016). Lepko et al. (2019) found that miR‐204 present in EVs that originate from the choroid plexus are taken up by NSCs of the sub‐ventricular zone (SVZ) and maintain them in the quiescent state but primed for rapid neurogenesis (Lepko et al., 2019). Apparently, an EV‐based long‐distance signalling pathway regulates the number of quiescent NSCs in the SVZ.

In the adult mammalian brain, two NSC niches directly contact CSF and may thus have access to CSF‐born EVs (Figure 1). By far the best‐studied NSC niche is the SVZ that contains neural progenitors some of which (B1 cells) project a primary cilium into the lateral ventricle (Doetsch et al., 1999; Obernier & Alvarez‐Buylla, 2019; Seri et al., 2004). A second niche lies in the posterior part of the ventral third ventricle (v3V, Figure 1), in a region populated by tanycytes. They project a primary cilium into v3V and have a single, long basal process that contacts distinct nuclei of the hypothalamus (Yoo & Blackshaw, 2018). A sub‐set of tanycytes shows proliferation and neural differentiation in the postnatal rodent brain (Haan et al., 2013; Horiguchi et al., 2020; Pellegrino et al., 2018; Robins et al., 2013).

The CSF flow in the v3V (Faubel et al., 2016) could deliver EVs secreted by the choroid plexus to the stem cells (Faubel et al., 2016; Sawamoto et al., 2006). A direct link between CSF‐born factors and NSC development and differentiation was seen in embryonic brain development (Chau et al., 2015; Feliciano et al., 2014; Lehtinen et al., 2011; Zappaterra & Lehtinen, 2012) and also in the adult brain (De Sonnaville et al., 2020; Kokovay et al., 2012; Lepko et al., 2019; Silva‐Vargas et al., 2016). Several studies indicated a direct connection between EVs present in CSF and NSCs niches (Batiz et al., 2015; Feliciano et al., 2014; Lepko et al., 2019; Losurdo & Grilli, 2020; Lun et al., 2015; Willis et al., 2022; Zhang et al., 2017). Yet much needs to be learned about the chemical identity of EV‐born neurogenic factors, how they are packaged into EVs, how they are delivered to and interact with the NSCs and which responses they evoke in their targets. We have developed an in vitro assay that can address some of these questions. We prepared EVs from the secretome of the rat Z310 choroid plexus cell line (EV^Z310^), by a combination of differential centrifugation and flotation on an iodixanol density gradient (Crescitelli et al., 2020; Kowal et al., 2016). These EV^Z310^ were co‐cultured with NSCs isolated either from the SVZ or from the tanycyte region. EVs were added to NSCs in medium or, alternatively, as dried‐down deposits. In both cases, purified EVs^Z310^ induced the compact rounded NSCs to rapidly form intricate cellular networks in which multiple cells were contacting each other through long processes. EVs produced by choroid plexus primary culture evoked NSC differentiation in the similar manner. These dramatic morphological changes were accompanied by the induction of genes in the differentiating cells, genes that are characteristic of early neurons and astrocytes. Gene induction was dose‐dependent and reached saturation at EV^Z310^ concentrations of 1.2–1.5 × 10^9^ particles per millilitre. LC‐MS/MS showed that the differentiation‐inducing EV^Z310^ were enriched for membrane and membrane‐associated proteins involved in cell differentiation, membrane trafficking and membrane organization. EVs purified from mouse embryonic fibroblasts (EV^MEF^) had little effect on the NSCs and did not show an enrichment of membrane and membrane‐associated proteins.

## 2 MATERIALS AND METHODS

A list of the antibodies and primers, which were used, is provided in the Supplementary information.

### 2.1 Cell culture

Immortalized Z310 rat choroidal epithelial cells were used as a choroid plexus substitute. Zhang et al. established the Z310 cell line in 2002 (Zheng & Zhao, 2002), starting with choroid plexus tissue collected from Sprague–Dawley rats (4–6 weeks old, both sexes). Such Z310 cells have been used in multiple studies, including those in which the choroid plexus primary culture and Z310 cells were compared side by side and were shown to be similar (Shi et al., 2008; Szmydynger‐Chodobska et al., 2007). Based on this, various groups used Z310 cells as a choroid plexus primary culture substitute (Grapp et al., 2013; Hasselblatt et al., 2009; Kläs et al., 2010). MEFs, were isolated from 13.5‐day‐old C57BL/6N mouse embryos. The choroid plexus contains a small number of fibroblasts (in addition to immune cells and perivascular cells) (Dorrier et al., 2022) and thus using EVs from a cell type that also occurs in the choroid plexus, provides a control. After the removal of head, liver and heart, embryos were cut into small pieces which were digested with 0.05% trypsin‐EDTA (Gibco) for 30 min at 37°C. The trypsin was inactivated by the addition of culture medium (see below), the cell suspension was pipetted repeatedly with a P1000 pipette and centrifuged for 5 min at 200 × *g*. Cell viability and count were determined by flow cytometry. Both Z310 and MEF cells were grown in DMEM supplemented with 10% FBS, 1x GlutaMAX and 50 units/Penicillin/Streptomycin (all Gibco) in a humidified incubator with 95% air, 5% CO_2_ at 37°C. They were passaged twice a week and were regularly checked for Mycoplasma contamination.

### 2.2 Purification of EVs by differential centrifugation

**FIGURE 2:**
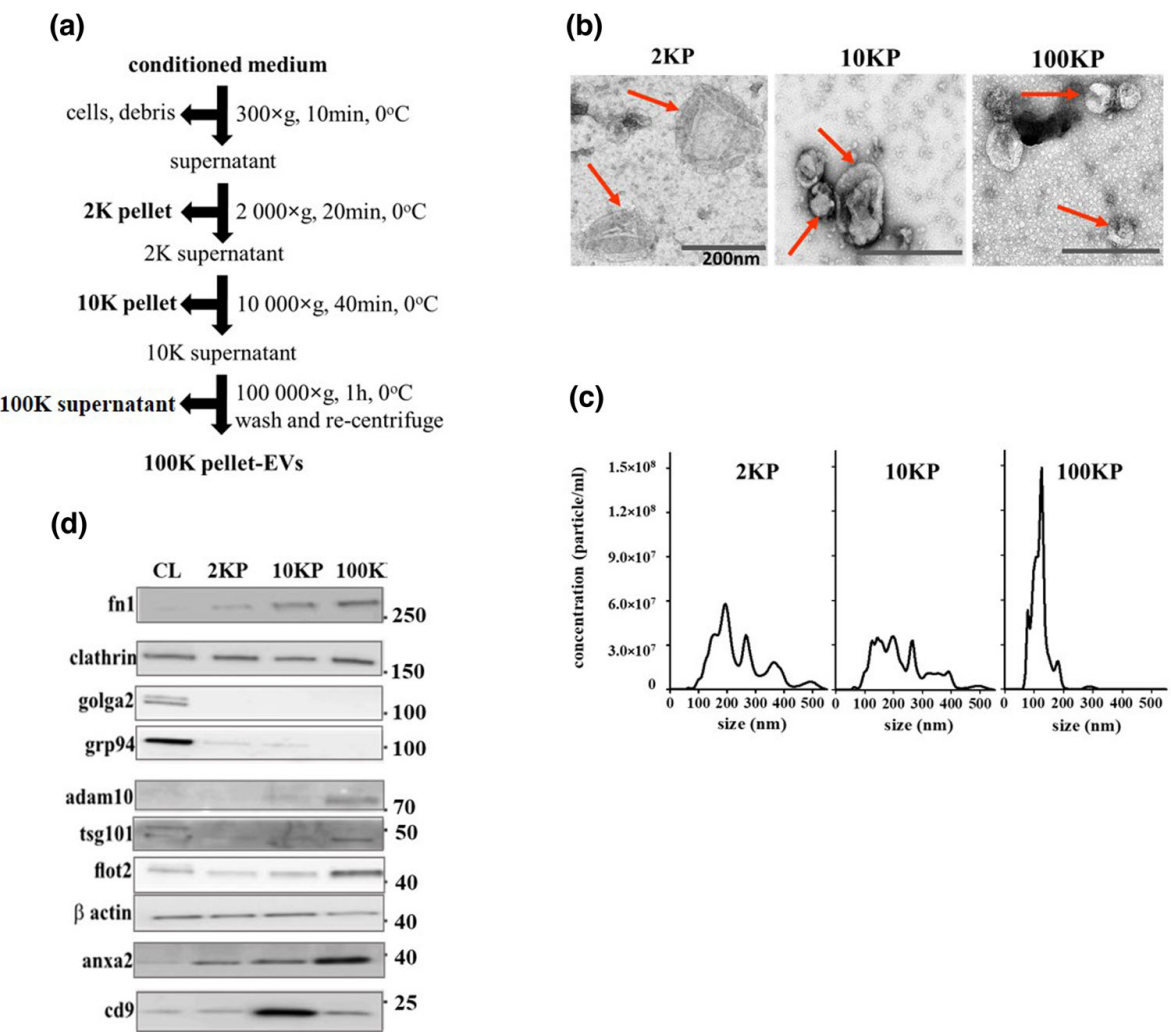
Purification and characterization of EVs from Z310 cell‐conditioned medium. (a) Scheme of purification of EVs from Z310 or MEF‐conditioned medium by differential centrifugation. (b) Representative electron micrographs of Z310‐conditioned medium‐derived vesicles (red arrows) present in the 2K, 10K and 100K Z310 pellets indicated in the scheme of Figure 2a. (c) Nanoparticle tracking analysis data showing the size distribution of the particles isolated from Z310‐conditioned medium in the various pellets that are indicated in the purification scheme of Figure 2a. The 100K pellet contains EV^Z310^ in a size range of 50–150 nm. (d) Individual fractions obtained in different steps of purification of Z310 supernatant were analysed by Western blotting. Z310 cell lysate (CL) is the lane on the left.

Cells with a confluency of 70%–80% were washed twice with PBS. To exclude carrying along EVs from serum, Z310 cells and MEFs were maintained for 2 days in serum‐free conditioned medium. The medium was then aspirated without disturbing the cells and subjected to differential centrifugation, as described by Kowal et al. (2016) (Figure 2a). The percentage of live cells at the time of harvesting was determined by Trypan Blue (Sigma). The cell pellet was collected and resuspended in 0.4% (v/v) Trypan Blue. Dead and live cells were counted using a hemocytometer (Nexcelon, Bioscience). Greater than 90% of the cells were viable, for example, in the case of Z310 cells, viability was 92.5± 2.1%. Centrifugation steps (Figure 2a): 300 × *g* for 10 min to remove cells and cell debris, then at 2000 × *g* for 20 min (Eppendorf 5702R) and 10,000 × *g* for 40 min (Eppendorf 5417R) to remove larger vesicles. The 10K supernatant was concentrated at 0°C using Vivaspin (300000 MWCO, Sartorius, Göttingen, Germany). This concentrated supernatant was centrifuged at 100,000 × *g*, for 60 min (Sorvall WX‐Ultra 80, Thermo Fisher Scientific, USA) in a TH‐660 rotor (Thermo Fisher Scientific). The resulting pellet was re‐suspended in ice‐cold PBS and centrifugation was repeated under the same condition. The resulting pellet was re‐suspended in PBS and stored at −80°C.

### 2.3 Size distribution and particle concentration

EV size and concentration were determined using a nanoparticle tracking analyzer (Nanosight NS300, Malvern Panalytical, Kassel, Germany). Five 60s videos with more than 200 tracks were taken per sample and analysed using the Nanoparticle Analysis software. Results represent the mean of all five measurements per sample.

### 2.4 Transmission electron microscopy analysis

The 2KP, 10KP and 100KP pellets were visualized by negative staining. Samples were adsorbed on the surface of a carbon‐coated copper grid and stained with 1% uranyl acetate. Grids were imaged by transmission electron microscopy, using a Talos L120C instrument (Thermo Fisher Scientific, Netherlands) equipped with a CMOS camera.

### 2.5 Western blotting

Eighty percent confluent Z310 cells were washed twice with PBS and lysed in lysis buffer (25 mM Tris‐HCl pH 7.4, 150 mM NaCl, 1 mM EDTA, 1% NP‐40 und 5% glycerol) according to the manufacturer's protocol, supplemented with a protease inhibitor cocktail (Halt^TM^ Protease and Phosphatase inhibitor cocktail) (all Thermo Fisher Scientific). The protein concentration of each pellet and the cell lysate was determined using a BCA kit (Pierce^TM^, Thermo Fisher Scientific). Equivalent quantities of proteins in pellets or in cell lysates were mixed with SDS loading buffer (1M Tris pH 6.8, 4% SDS, 20% glycerol, 10% mercaptoethanol, 0.2% bromophenol blue) heated at 95°C for 10 min and loaded on a 4%–20% polyacrylamide gradient gel (Bio‐Rad Laboratories). Subsequently, samples were transferred to a PVDF membrane (Bio‐Rad Laboratories). Membranes were blocked with 5% non‐fat dry milk powder in TBST (0.1% Tween 20 in Tris‐buffer saline) for 30 min, followed by overnight incubation with the appropriate primary antibody, at 4°C. After washing, membranes were incubated with the secondary antibody for 1 h at RT. All antibodies were diluted in blocking buffer according to the manufacturer's recommendations. After washing, membranes were developed using ECL Super signal West Femto (Thermo Fisher Scientific), and protein bands were visualized on an Image Quant LAS 4000 imager (GE Healthcare Bio‐science AB, Sweden).

### 2.6 Sample preparation for LC–MS/MS

Isolated EVs were lysed by adding an equal volume of lysis buffer (4% [wt/v] SDS 1 mM EDTA 100 mM Hepes, pH 8) supplemented with a protease inhibitor cocktail (cOmplete, Roche) and sonicated in a BioRuptor (Diagenode) with 30s on‐and‐off cycles for 5 min. Proteins were reduced, alkylated and purified as described (Hughes et al., 2014; Silbern et al., 2021). The digestion was accomplished overnight in 50 mM ammonium bicarbonate buffer using trypsin (Promega)‐to‐protein ratio (wt/wt) of 1:20. Vacuum‐dried samples were re‐dissolved in 2% (v/v) acetonitrile 0.1% (v/v) trifluoroacetic acid in water and subjected to LC–MS/MS analysis.

### 2.7 LC–MS/MS acquisition

Samples were analysed using an Orbitrap Fusion Tribrid or a Q‐Exactive HF‐X (both Thermo Fisher Scientific) mass spectrometer interfaced via an LC set‐up as described (Silbern et al., 2021). Peptides were separated using 118 min linear gradients ranging either (A) from 4% to 5.6%, 16%, 25.6%, 40%, 90% and back to 4% (v/v) acetonitrile over 3, 57, 30, 16, 6 and 6 min, respectively (Orbitrap Fusion) or (B) from 1.8% to 4%, 33.6%, 72% and back to 1.8% over 5, 101, 6, and 6 min (Orbitrap HF‐X). The mass spectrometer was operated in a data‐dependent acquisition mode. A survey scan was performed at 120,000 resolution and 50 ms maximum injection time (MaxIT). Thirty top‐abundant peptide precursors (or keeping a constant duty cycle of 3 s) were selected for sequencing using a 1.6 *m/z* isolation window. Precursor ions were fragmented in an HCD cell and the normalized collision energy setting of 28% or 30%. MS/MS spectra were acquired in Orbitrap operated using 15,000 resolution and 54 ms MaxIT or 30,000 resolution and 120 ms MaxIT. Precursors were excluded from repeated sequencing for 30 s.

### 2.8 LC–MS/MS data analysis

Raw MS data were processed by MaxQuant (version 1.6.2.10) (Cox et al., 2011) using default parameters. MS/MS spectra were searched against *Rattus norvegicus* and *Mus musculus* canonical protein sequences from Uniprot (Bateman, 2019) (December 2020, 29,940 and 17,051 sequences, respectively). Protein groups with at least two razor or unique peptides were considered as identified. IBAQ values (Schwanhäusser et al., 2011) attributed to the same gene name but several protein groups due to different origin (rat/mouse) were summed in order to achieve a single quantitative value per gene. In the following, only genes quantified in at least two EV^Z310^ replicates were considered for further analysis: iBAQ values were log2‐transformed, normalized by median‐subtraction, and missing values were imputed by random sampling from a normal distribution centred at the 5%‐intensity quantile and a standard deviation equal to a half of the standard deviation of each replicate's intensities. *Limma* R package (Smyth, 2005) was used to test for differential protein abundance in EV^Z310^ and EV^MEF^. Candidates satisfying the criteria of *q*‐value < 0.01 (Storey, 2002) and an absolute log_2_FC > 1 were considered as differentially expressed.

### 2.9 GO pathway enrichment analysis and protein–protein interaction network functional enrichment analysis

Proteins identified in EVs by MS were analysed by DAVID (Database for Annotation, Visualization and Integrated Discovery). Proteins identified in EV^Z310^ belonging to the cellular component category ‘vesicle’ were used as input for STRING (protein–protein interaction networks functional enrichment analysis). To visualize functional interactions, MCL clustering was used. To contrast EV^Z310^ and EV^MEF^ proteomes, the GO category ‘cellular component’ was selected after functional annotation clustering. For each cellular component, the Benjamini‐corrected *p*‐value was displayed as bar graph, from the highest ranking to the lowest one. Analysis revealed main differences in sub‐category ‘membrane’. Proteins present in EV^Z310^ but not in EV^MEF^ were used as input for the subsequent STRING analysis.

### 2.10 Iodixanol density gradient centrifugation

Flotation in an iodixanol gradient was performed as described in Crescitelli et al. (2020) with some modifications. The 100K pellet obtained after differential centrifugation was re‐suspended in 1 mL of 40% iodixanol (v/v) (OptiPrep^TM^ Density Gradient Medium, Sigma) in PBS and bottom loaded. For the discontinuous iodixanol gradient equal volumes of solutions of 30%, 20% and 10% iodixanol were layered on the top of the sample (Figure 6a) and centrifuged in a 4‐mL tube (Beckman Coulter, 328874) at 180,000 × *g* for 19h, at 0°C (Sorvall WX‐Ultra 80, Thermo Fisher Scientific) in a TH‐660 rotor (Thermo Fisher Scientific). Fractions of 500 μL from top to bottom were collected from the tube. An opaque band of EVs was recovered in fraction 6.

### 2.11 NSC dissection and neurosphere expansion

Animal experiments were carried out in accordance with regulation of the Office of Consumer Protection and Food Safety of Lower Saxony and conformed to German Laws on Animal Welfare. To isolate NSCs from SVZ of the lateral ventricle and the tanycyte region of v3V, 6–8 weeks old male mice of the strain C57BL/6N were sacrificed. To isolated stem cells from the tanycyte area of the v3V, intact brain was placed in a brain matrix (Plano, GmbH) sub‐merged in HBSS (Hank´s balanced solution with Ca and Mg, Gibco) supplemented with glucose (0.45%). Two razor blades were used to cut 3 mm thick coronal sections. Under the stereomicroscope, v3V was isolated and the tanycyte area was separated, using tungsten needles (for details see Faubel et al. (2016)). The NSCs from SVZ of the lateral ventricle were isolated according to Walker and Kempermann (Walker & Kempermann, 2014). SVZ and tanycyte tissues were minced with a scalpel blade, transferred to pre‐warmed 0.05% Trypsin‐EDTA and incubated for 10 min at 37°C with mixing every 3 min. Then the tissue was dissociated by gentle pipetting. The enzymatic reaction was stopped by mixing with pre‐warmed trypsin inhibitor (0.125 mg/mL containing DNase I, 0.01 mg/mL), then spun down for 5 min at 300 × *g*. The pellet was re‐suspended in medium and spun again. The final pellet was re‐suspended in complete growth medium (Neurobasal Medium with supplement, see below) and passed through cell strainers (first 70 μm, then 40 μm) and placed into an ultra‐low attachment surface 6‐cm polystyrene dish (Corning, USA). After 7 days, neurospheres were visually inspected. The growth medium was changed every second day.

### 2.12 Dissociation of neurospheres

The NSCs were grown in Neurobasal Medium supplement with 2% B27, 1% N2 supplement, 1x GlutaMAX, 50 units/mL Penicillin/Streptomycin (all Gibco), 20 ng/mL purified mouse receptor‐grade epidermal growth factor (EGF), and 20 ng/mL recombinant bovine fibroblast growth factor (FGF‐2) (both Peprotech). Neurospheres were passaged by centrifugation at 300 × *g* for 5 min. The pelleted spheres were re‐suspended in prewarmed 0.05% Trypsin‐EDTA and incubated at 37°C for 5 min, then an equal volume of trypsin inhibitor (0.125 mg/mL containing DNase I, 0.01 mg/mL) was added. The resulting individual cells were centrifuged for 5 min at 300 × *g*, and the cell pellet was resuspended in fresh medium. The cells were counted using a hemocytometer and seeded at a concentration 2 × 10^5^ cells/mL for all experiments.

### 2.13 Choroid plexus primary culture

To isolate choroid plexus from the lateral ventricle, 6–8 weeks old male mice (C57BL/6N) were sacrificed. Isolation and culture of primary mouse choroid plexus were performed essentially as previously described (Menheniott et al., 2010). Choroid plexus was removed from the lateral ventricle at ZT 5, and rinsed in HBSS supplemented with glucose. Thereafter, the HBSS was aspirated, pre‐warmed Pronase (2 mg/mL) was added, followed by incubation at 37°C for 5 min. Growth medium was added to stop digestion, the cells were centrifuged for 5 min 300 × *g*, the supernatant was removed and the cell pellet was suspended in fresh medium. Another step of centrifugation followed (5 min 300 × *g*). The final pellet was suspended in complete DMEM growth medium (10% FBS, 50 units/mL Penicillin/Streptomycin, 10 ng/mL EGF) supplemented with 20 μM cytosine arabinose and plated on poly‐D‐lysine coated plates which were kept in a humidified incubator (95% air, 5% CO_2_) at 37°C. The medium was changed every 48 h until cells became fully confluent.

### 2.14 Differentiation assay with EVs in suspension

NSCs in neurobasal medium were seeded on an appropriate size petri dish (Thermo Fisher Scientific, Denmark, 153066) and EVs from the 100K pellets, re‐suspended in PBS were added. Amounts of added EVs were normalized to their total protein concentration, as determined by a BCA assay. The NSCs were cultured in a CO_2_ incubator for 24 h and bright field images of live cells were taken. After photography, the NSCs were processed for immunohistochemistry, qPCR or flow cytometry.

### 2.15 Differentiation assay using EV dry drops

EVs were applied on the surface of the eight‐chamber tissue culture slide (Corning, USA, 354108) as a 1‐μL drop. The drop was dried (approximately 15 min) and then NSC (2 × 10^5^ cells/mL) in culture medium were added and chambers were placed into a CO_2_ incubator. Twenty‐four later images of the chamber wells were taken. For the testing of stability of dried‐down EVs, drops were applied to the wells of the eight‐chamber slides which were then kept for 7 days at −20°C.

### 2.16 Protease and nucleases protection assay

Proteinase K (Bioline) was added to a final concentration of 10 μg/mL to the EVs. Samples were incubated for 30 min on ice. Proteinase K was inactivated for 10 min by incubating with tetrapeptidyl chloromethyl ketone at a final concentration of 10 μg/mL (EMD Millipore) on ice. Then DNase (2U final concentration) was added and the sample was incubated at 37°C for 30 min. The reaction was stopped with DNase inhibitor (2U final concentration) (DNA free kit, DNase treatment and removal, Invitrogen, Thermo Fisher Scientific). Then RNase A (40U final concentration, 37°C, 30 min) (Qiagen) was added and inactivated by RNase inhibitor (40U/mL) (Invitrogen, Thermo Fisher Scientific). Samples were cleaned using an Amicon ultra centrifuge unit (Merck, Millipore) with PBS. The resulting EVs^Z310^ were then added to NSCs^tz^ or NSCs^SVZ^ for assessing their differentiation inducing capacity.

### 2.17 Immunofluorescence

Cells on slides or in Petri dishes were fixed with 4% formaldehyde, washed with PBS (0.1% Triton) and blocked for 30 min (0.25% Triton and 0.25% BSA in PBS) at room temperature. Then cells were incubated overnight at 4°C with primary antibody. After the three washing steps with PBS (0.1% BSA, 0.1% Triton), incubation with secondary antibody for 1 h at room temperature followed. After five washes, coverslips were mounted with mounting medium with DAPI (Vectashield, Vectorlabs) and images were acquired using a Leica DMI 6000B fluorescence microscope, with LAS X software (Leica Microsystems, Germany).

### 2.18 RNA preparation for qPCR analysis

Total RNA was extracted from cells using Trizol reagent (Ambion) or RNeasy kit (Qiagen) according to the manufacturer´s instructions. First genomic DNA was removed and then 1 μg RNA was reverse transcribed by iScript gDNA Clear cDNA synthesis Kit (Bio‐Rad Laboratories) according to manufacturer's instructions. *Gapdh* was used for normalization. qPCR was performed with SsoAdvanced Universal SYBR Green Supermix (Bio‐Rad Laboratories) on CFX96‐Real‐Time system (Bio‐Rad Laboratories, Germany). For primers, see Supplementary information, Material.

### 2.19 Flow cytometry

NSCs were analysed after 24 h of culturing in the presence of EVs. Single‐cell suspensions were obtained after accutase‐mediated (ESGRO Complete^TM^ Accutase, EMD Millipore) dissociation. Cells were then fixed with 4% formaldehyde for 15 min in the dark at room temperature. Washing and blocking steps were followed by overnight incubation with primary antibody at 4°C. After the washing steps, incubation with the appropriate fluorescent secondary antibody for 1 h at RT followed. The cells were analysed using a flow cytometer BD Accuri C6 (BD Bioscience, USA) and BD Accuri^TM^ C6 software.

### 2.20 Proliferation assay

NSCs were seeded on 96‐well flat‐bottom tissue culture plates of good optical quality (Falcon) at a density of 2 × 10^5^ cells per millilitre. Cells were cultured for 24 h in Neurobasal Medium (control) or in medium containing an increasing concentration of EV^Z310^ or EV^MEF^. Thereafter, MTT solution was added and all subsequent steps were performed according to manufactures’ instructions (Merck, Colorimetric (MTT) kit for cell survival and proliferation). Absorbance was measured with an ELISA plate reader (Infinite M2000 Pro Tecan) with a test and reference wavelength of 570 and 630 nm, respectively. For the Trypan Blue assay, NSCs were seeded on a 6‐cm Petri dish at a density of 2 × 10^5^ cells per millilitre and cultured in increasing concentration of EV^Z310^ or EV^MEF^ for 24 h. Trypan Blue staining was performed according to the manufacture’s instruction (Sigma) and the number of cells was determined with a hemocytometer.

## 3 RESULTS

### 3.1 Characterization of EVs and their protein composition

We purified EVs secreted by rat Z310 choroid plexus cells (EV^Z310^), by mouse choroid plexus primary culture (EV^CHP^) and by mouse embryonic fibroblasts (EV^MEF^). The Z310 cell line is a widely used surrogate for choroid plexus primary cells (for references for the Z310 cells usage see Materials and Methods). Conditioned media were collected and subjected to differential centrifugation (Figure 2a). Pellets obtained after each centrifugation step were analysed for particle size, particle concentration and the presence of EV protein markers (Thery et al., 2018) (Figures 2b–d and S1a–c). The 2K and 10K pellets had the highest total protein content and nanoparticle tracking analysis and electron microscopy revealed the presence of particles in the size range of 100–500 nm (Figure 2b,c). Centrifugation at 100,000 × *g* removed particles >200 nm and yielded predominantly particles of 100 nm, the size for exosomal EVs (Figures 2b,c and S1a,b). Choroid plexus primary culture cells expressed transthyretin (Figure S2a) and produced EVs in the size range of 30–120 nm (Figure S2b). Fibronectin 1 (Fn1), disintegrin and metalloproteinase domain‐containing protein 10 (Adam10) and tumour susceptibility gene 101 (Tsg101) are typical for small EVs (Tkach et al., 2018) and they were noticeably enriched in the 100K pellet (Figure 2d). Annexin A2 (Anxa2) and flotillin2 (Flot2) were elevated in this fraction but were also present in the 2K and 10K pellets. By contrast, endoplasmin (Gpr94, Hsp90b1), a marker for large vesicles (Tkach et al., 2018), was present in the whole cell lysate (CL), barely detected in the 2K and 10K pellets, and absent in the 100K pellet. Golgi marker, golgin sub‐family A member 2 (Golga2/Gm130) was found only in the cell lysate fraction (CL). *β* actin levels declined during purification, with a maximum in cell lysate and a minimum in the 100K pellet, for example, (Kowal et al., 2016) (Figure 2d). Figure S1c shows that in the 100K pellet containing EVs^MEF^, EV markers (fn1, clathrin, alix, tstg101 and flot2) were present. By contrast, golga2 and grp94 were absent, as expected, in the EVs^MEF^ and found in MEF cell lysate.

The 100K Z310 pellet obtained after the ultracentrifugation was subjected to mass spectrometric analysis. One thousand and two hundred proteins were identified (Table S1) which is in the range as reported for EVs isolated from mouse CSF (Balusu et al., 2016). The MS data confirmed the presence of the typical EV markers. Examples are transmembrane or GPI‐anchored tetraspanins (CD9, tetraspanin‐8), integrins (integrins alfa, ‐beta), basement membrane‐specific heparan sulphate proteoglycan core protein (Hspg2), basigin (Bsg), Adam10 and multidrug resistance‐associated protein 1 (Abcc1). Cytosolic proteins were also recovered in EVs including Tsg101, programmed cell death 6‐interacting protein (Pdcd6ip, Alix), vacuolar protein sorting‐associated protein (Vps4a), arrestin domain‐containing protein 1 (Arrdc1), Flot1/2, transforming protein RhoA (Rhoa), annexins (e.g., anxa1, ‐2, ‐5, ‐11), heat shock cognate 71kDa protein (Hspa8) and syntenin‐1 (Sdcbp). MS analysis also identified secreted protein typically recovered with EVs (e.g., lactadherin‐Mfge8). By contrast, neither cytokines (interleukins, interferons) nor growth factors were detected, with the exception of bone morphogenetic protein 1 (Bmp1). Bmp1, a metalloproteinase that was previously identified in the CSF and plays a role in the development of the CNS by stimulating progenitors in the SVZ (Lehtinen et al., 2011). Thus, Bmp1 could be one of the CSF‐born neurogenic factors that are transported inside EVs. Proteins associated with other intracellular compartments such as the nucleus (histones) or mitochondria (Tomm20) but not with Golgi apparatus (Golga2) were also detected. The 100K pellet also contained ribosomal proteins and proteasomes, large protein complexes known to co‐sediment with EVs (Konoshenko et al., 2018; Thery et al., 2018).

Gene ontology (GO) enrichment analysis of ‘cellular component’ categories showed that proteins identified in the 100K pellet were significantly enriched for ‘extracellular exosome’ ‘membrane’ and ‘vesicle’ sub‐categories (Figure 3a). Other enriched sub‐categories were ‘focal adhesion’, ‘adherent junction’, ‘extracellular matrix’, ‘neuron projection’, ‘neuronal cell body’, ‘dendrite’, ‘synapse’ and ‘axon’. Selected typical EV proteins retrieved from the sub‐category ‘vesicle’ formed a STRING protein interaction network (Figure 3b). We picked this sub‐category since it contained EV‐typical markers discussed above. Our analysis revealed a functional association and a significant number of interactions (PPI enrichment *p*‐value < 1.e^−16^). MCL clustering (inflation parameter 1.9) revealed four major proteins clusters (Figure 3b). The biggest cluster (red) included proteins associated with ‘SNARE, vesicle fusion and transport’. Two additional clusters (yellow, green) represented proteins involved in endosomal sorting complex required for transport (ESCRT), cell adhesion, morphogenesis and cellular projections. Proteins involved in ER‐Golgi transport formed the smallest cluster (blue). This result showed strong interactions of proteins relevant to EV biology.

FIGURE 3Analysis of EV^Z310^ and EV^MEF^ proteomes. (a) GO‐cellular components of EVs^Z310^. Note an enrichment in ‘extracellular exosome’, ‘membrane’, ‘vesicles’, ‘neuronal projection’, ‘neuronal cell body’, ‘dendrite’, ‘synapse’ and ‘axon’ sub‐categories. (b) Protein–protein interactions in EVs^Z310^ proteome for the gene ontology sub‐category ‘vesicle’, visualized by STRING. This indicates a functional interaction network. Solid lines inside clusters show direct physical protein interactions and dotted lines show functional interactions. (c) Venn diagram shows that EV^Z310^ and EV^MEF^ protein contents differ significantly. The diagram is based on combining three data sets (EV^Z310^) and two data sets (EV^MEF^) (see Supplementary Table 1). (d) GO‐cellular components of EV^MEF^. Comparison with EV^Z310^ shows differences in ranking of several categories such as ‘membrane’ that ranks high only in the EV^Z310^. (e) Functional analysis of protein–protein interactions by STRING. Proteins identified exclusively in EV^Z310^ and falling into the gene ontology sub‐category ‘membrane’ were included. Three main clusters of protein–protein interaction emerged.

**Figure:**
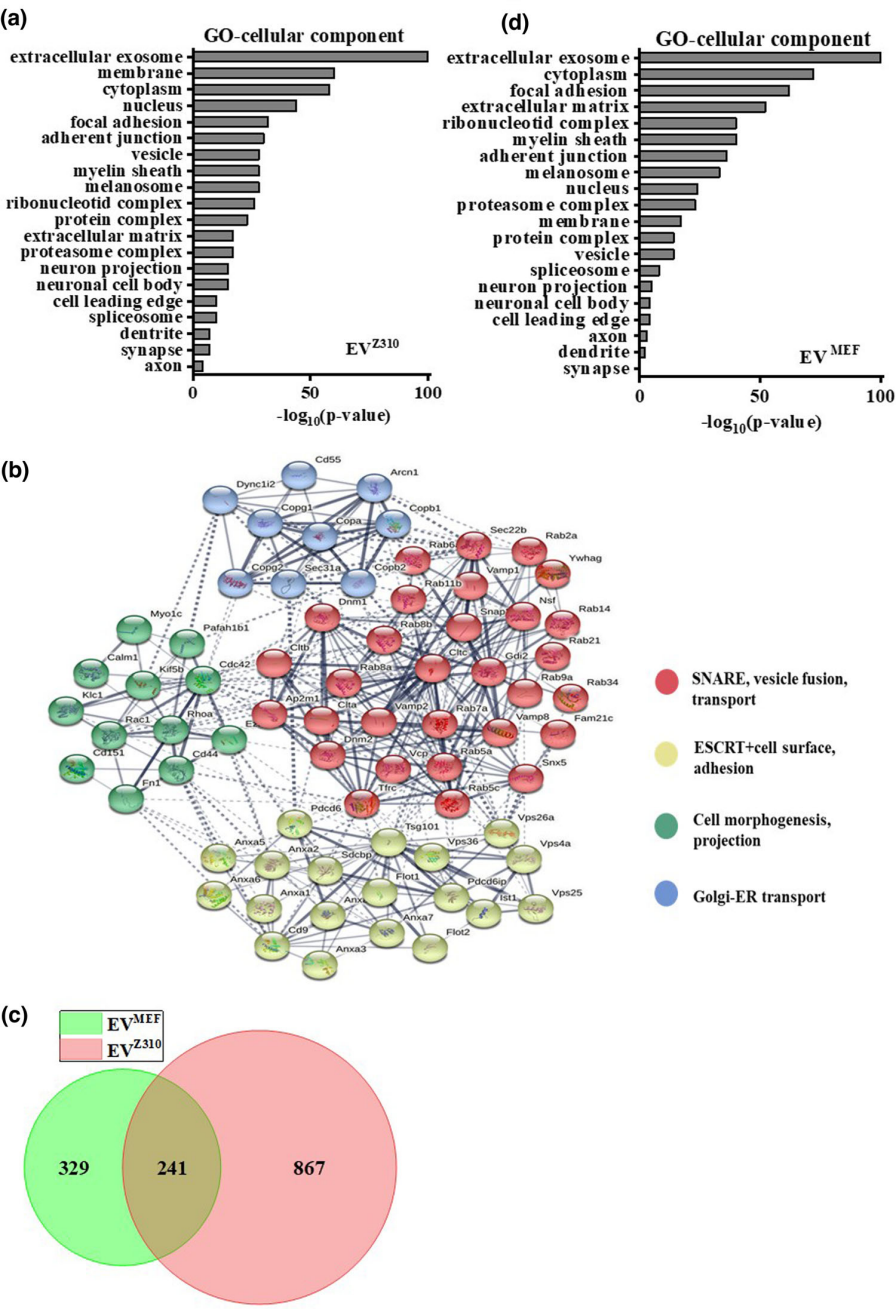

**Figure:**
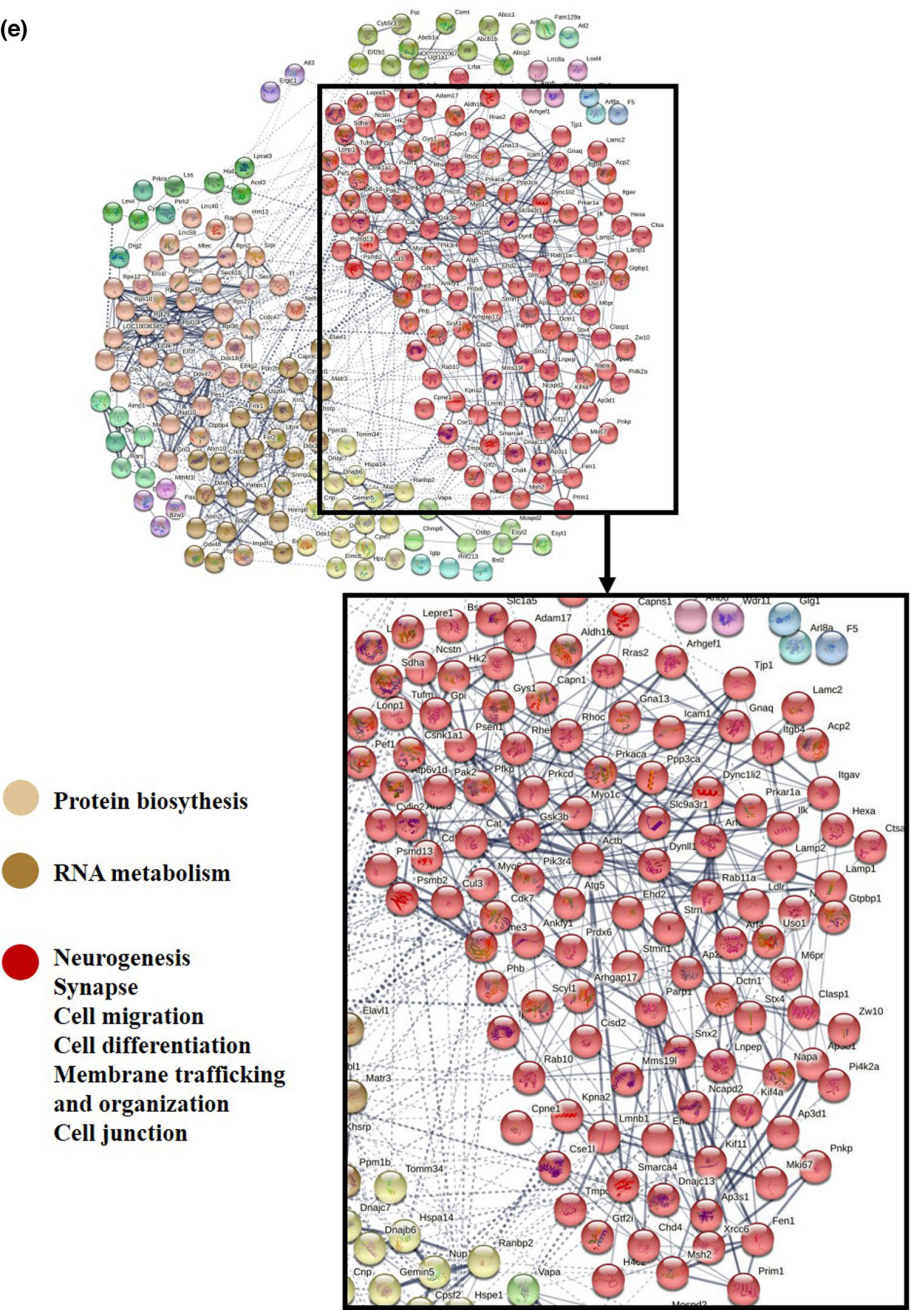

Western blots were used to investigate whether proteins identified by mass spectrometry were present as full‐length protein in EV^Z310^ lysates. We choose signal transducer and activator of transcription 3‐stat3 (92 kDa), slit homolog 2 protein‐slit2 (200 kDa) and transcription activator BRG1 also known as ATP‐dependent chromatin remodeler‐smarca4 (185kDa). Slit2 is a secreted extracellular matrix protein involved in axon guidance (Kaneko et al., 2018; Nguyen‐Ba‐Charvet & Chédotal, 2002; Sawamoto et al., 2006). Stat3 is a transcription factor and smarca4 is a transcriptional activator that both regulate GFAP expression (Brenner & Messing, 2021; Ito et al., 2018). Figure S1d demonstrates that these proteins are present in EV^Z310^ and electrophorese, according to the band size provided by the manufacturer.

EVs from MEFs (EV^MEF^), were prepared and analysed as described for EV^Z310^ (Figures 2a and S1a–c) and underwent MS analysis. Five hundred and seventy proteins could be identified (Table S1), less than for EV^Z310^. The likely cause for this difference is that Z310 cells but not MEFs are secretory cells. A Venn diagram (Figure 3c) reveals that ∼40% of the EV^MEF^ proteins were also present in EV^Z310^. Three hundred and twenty‐nine proteins were exclusive for EV^MEF^ and 867 for EV^Z310^. GO enrichment analysis for the category ‘cellular component’ showed in EV^MEF^ a significant enrichment of proteins in sub‐categories ‘focal adhesion’, ‘extracellular matrix’ and ‘adherent junction’. This finding is consistent with a role of fibroblasts in the CNS including the choroid plexus to provide structural support by secretion of extracellular matrix proteins (Dorrier et al., 2021). The sub‐category ‘membrane’ was much less enriched in EV^MEF^ than in EV^Z310^ and just a few EV^MEF^ components fell into the sub‐categories related to neurons.

STRING functional analysis of the 248 EV^Z310^ proteins that were classified into the sub‐category ‘membrane’ and not identified in EV^MEF^, yielded three prominent clusters (Figure 3e). Two small clusters (brown) compiled proteins involved in ‘RNA metabolism’ and ‘protein biosynthesis’. Examples are 60S ribosomal proteins L14 (Rpl14), Rpl27, Rpl36 and eukaryotic translation initiation factors (Eif4g2, Eif3k). Some of the ribosomal proteins that are present in 100K pellet co‐purify with EVs (Kowal et al., 2016) may not be EV cargo (Thery et al., 2018). The red cluster comprised proteins associated with ‘neurogenesis’, ‘cell differentiation’, ‘cell migration’, ‘membrane organization and trafficking’. Examples are Adam17 and a Rheb‐Ras homolog enriched in brain where it plays a role in neural plasticity. Cdk5, which is also in the red cluster, is important for neural migration and CNS development and regulates cytoarchitecture, axon guidance and membrane transport (Dhavan & Tsai, 2001). Proteins involved in sub‐category ‘synapse’ are sorting nexin‐4 (Stx4) and N‐ethylmaleimide‐sensitive factor attachment protein alpha (Napa). Examples of the proteins found in sub‐category ‘cell migration’ and ‘membrane organization’ are integrins, Itgb4 and integrin‐linked protein kinase‐Ilk, which are involved in cell adhesion, cell architecture and cell motility. Proteins of the ‘membrane trafficking and organization’ sub‐categories are Ras‐related protein Rab‐10 (Rab10), EH domain‐containing protein 2 (Ehd2), sodium‐hydrogen antiporter 3 regulator (Slc9a3r1). We took the composition of the red cluster as an indicator that EV^Z310^ contains proteins that could control the NSC biology.

### 3.2 EVs from the Z310 cell line induce NSC differentiation in a dose‐dependent manner

As shown by MS analysis, EVs from the choroid plexus‐derived Z310 cell line contain many proteins associated with membrane function and neuronal differentiation (Figure 3). It is possible that after their secretion by the choroid plexus into the CSF, some EVs are transported to the NSC niches (Figure 1) where they might affect NSC membrane and/or evoke the differentiation of the NSCs. To test this hypothesis, we prepared NSCs from the tanycyte region and the SVZ of mouse brain (Figure 1) and generated NSC aggregates (neurospheres) using standard methods (see Materials and Methods and Walker and Kempermann (2014)). In accordance with previous work (Haan et al., 2013; Robins et al., 2013; Walker & Kempermann, 2014), the NSCs from either region formed neurospheres (Figure S3a), that expressed typical NSC markers, such as *nestin*, *paired box protein 6 (Pax6), Gfap, Sox2* and *vimentin* (Figure S3b).

**FIGURE 4:**
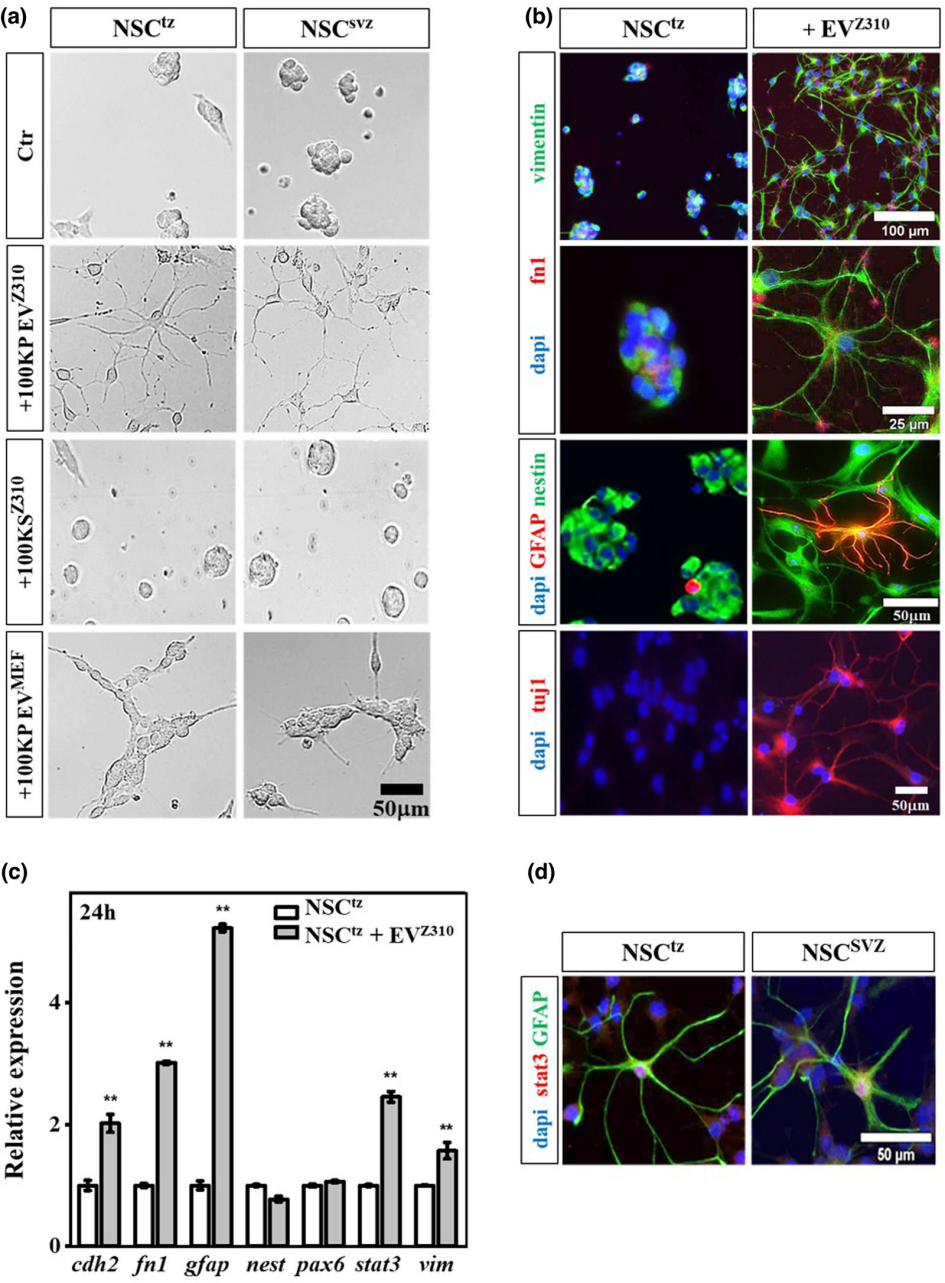
EV^Z310^ promote NSC differentiation into cellular networks. (a) Bright‐field images of NSC^tz^ and NSC^SVZ^ co‐cultured for 24 h with medium only (Ctr), with EV^Z310^, with Z310 100K supernatant (100KS^Z310^) or with EV^MEF^. Total EV protein concentration was 76 μg/mL. (b) Immunostaining for various markers visualized cell structure and cell processes that formed in the presence of EVs^Z310^ (24 h). Astrocytes (GFAP) and early neurons (Tuj1, 48 h) were seen. NSCs cultured in medium without EVs^Z310^ did not form processes, but just spherical aggregates of an irregular shape. Note the punctate fn1 staining in the differentiating EV^Z310^‐treated NSC^tz^. (c) qPCR showed upregulation of *cadh2, fn1, gfap, stat3* and *vimentin* expression 24 h after EV^Z310^addition. Values are presented as means ± SD, three biological and three technical replicates were done (**p < 0.01, Student’s *t* test). (d) Immunofluorescence staining for astrocyte marker (GFAP) showed astrocytic cells with multiple processes and positive staining for nuclear Stat3 in NSC^tz^ and NSC^SVZ^ after a 24‐h co‐culture with EVs^Z310^.

After the neurospheres were dissociated into single cells, EVs^Z310^ were added to them. We found that within 24 h, the NSCs from the tanycyte niche (NSC^tz^) formed complex cellular networks in which individual cells cross‐connected with each other through long processes (Figure 4a, first column, second row). NSCs from the SVZ niche (NSC^SVZ^) yielded similar cellular networks (Figure 4a, second column, second row). In the case of EV^Z310^‐exposed NSCs^tz^, 50 ± 2% of the attached cells were in contact with at least one neighbour and had ≥3 processes that were ≥ 20‐μm long. In the case of NSCs^SVZ^, this percentage was 50 ± 5%. Therefore, network‐forming cells account for half of all attached cells. We rarely saw such processes in controls in which only culture medium was added to NSCs (Figure 4a, top row). Likewise, EV^MEF^ evoked clustering of just a few NSCs^tz^ or NSCs^SVZ^ and we did not observe a cell process‐based network (Figure 4a, bottom row). The EV‐free 100K supernatant from the differential centrifugation step (Figure 2a) added to NSCs^tz^ or NSCs^SVZ^ had no effect on these cells (Figure 4a, third row).

Immunostaining of fixed NSC^tz^ cultures with antibodies against intermediate filament proteins vimentin and nestin visualized the fine structure of the cellular processes and showed that processes originating from a particular cell can make multiple contacts with the neighbouring cells (Figure 4b, right column). Note, the glial fibrillary acidic protein (GFAP) expressing cell (Figure 4b, right column, third row), probably an astrocyte, contacts multiple times with the nestin‐expressing cellular network. Network‐forming cells account for half of all attached cells and the GFAP‐positive cells belong chiefly to this group of attached, stellate cells. Single cells with a compact morphology (presumably residual NSCs) express GFAP at very low levels in the cytoplasm surrounding the nucleus (Figure 4b, first column, third row). The extracellular matrix protein fibronectin1 (fn1) co‐localized with the tip of the processes (Figure 4b, right column, second row). Tuj1 (β3 tubulin) staining for early neurons revealed groups of interconnected cells (Figure 4b, right column, bottom row). Figure 4d shows staining for Stat3 protein in the nucleus of the GFAP‐expressing astrocytes. Stat3 activates *Gfap* expression (Brenner & Messing, 2021; Ito et al., 2018). qPCR analyses showed that NSC morphological changes were accompanied by a significant transcriptional upregulation of the genes encoding for *fn1, stat3*, *vimentin, gfap* and also of the neuronal cadherin, *cadherin‐2* (*Cdh2)* (Figure 4c).

EVs^CHP^ produced by choroid plexus primary cells had a similar effect on NSCs as EVs^Z310^. Both induced cellular network formation and cell differentiation (Figure S2c,d). Our data suggest that EV^Z310^ can substitute for primary culture‐derived EVs.

Since this study focusses primary on the early response of NSCs to EVs, the expression data we present cover, for the most part, the first 24 h after adding EV^Z310^. As discussed below, during this period, the effect of the EVs on cell proliferation is very limited (Figure S4c–e). The percentage of cells expressing Tuj1 or GFAP is rather small by 24 h, but by 48 h, both proteins show a modest increase, at least, when a high dose of EVs^Z310^ was added. After 24 h, the fraction of Tuj1 or GFAP‐expressing cells was 0.5% and 10%, respectively (304 μg/mL; Figure S4a,b). At 304 μg/mL, of EVs, and 48h of incubation, these percentages increased to 5% (Tuj1) and 12%–15% (GFAP), respectively. Previous work (Silva‐Vargas et al., 2016) found that after 7 days of incubation with choroid plexus secretome, the corresponding percentages were 15% for neurons and 80% for astrocytes. Evidently, providing the entire choroid plexus secretome for a longer time leads to more pronounced differentiation of NSCs than seen with a short treatment with Z310‐derived EV^Z310^.

**FIGURE 5:**
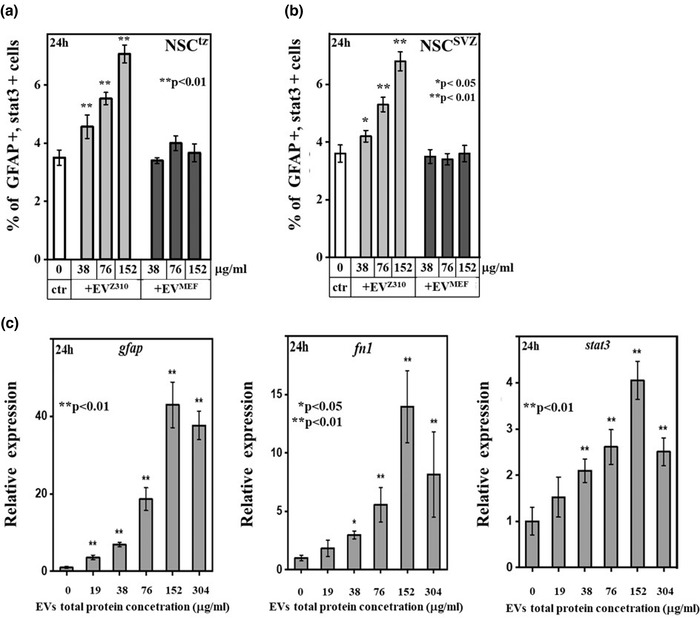
EV^Z310^ promote NSC differentiation in a dose‐dependent manner. (a) Flow cytometry showed that after 24 h EV^Z310^ increased the number of GFAP‐ and Stat3 positive NSCs^tz^ in a dose‐dependent manner (total protein concentration 38, 76, 152 μg/mL). In contrast, EV^MEF^ did not elevate the number of GFAP‐ and Stat3‐positive cells above the control at all concentrations used. (b) Such a dose‐dependent increase was also seen when NSCs^SVZ^ were co‐cultured with EVs^Z310^. (c) qPCR graphs demonstrated a dose‐dependent increase of *gfap*, *fn1* and *stat3* mRNA 24 h after adding EVs^Z310^ to NSCs^tz^. Values are means ± SD from three independent experiments (*p < 0.05, **p < 0.01, Student’s *t* test).

Flow cytometry was used to quantify the expression levels for GFAP and Stat3 proteins after coincubation of NSCs^tz^ or NSCs^SVZ^ with increasing doses of EVs (Figure 5a,b). The fraction of GFAP/stat3 ^+^ cells increased with increasing amounts of EVs^Z310^ (assessed by total protein content) added to the culture. When using EV^MEF^, the fraction of GFAP/stat3 ^+^ cells remained at control levels (Figure 5a,b). The flow cytometry data are fully consistent with transcript quantification by qPCR. Levels of *gfap, fn1* and *stat3* expression showed a dose‐dependent upregulation upon EV^Z310^addition. Expression reached a saturation (Figure 5c) and the 50% effective dose (ED_50_) was in the range of 50–70 μg/mL EV protein.

CSF from the cisterna magna, and the choroid plexus secretome induced the proliferation of NSCs (Silva‐Vargas et al., 2016). This research as well as work by other authors (Lehtinen et al., 2011) focusses on NSC treatments with the inducing agent for 7 days, which is well beyond the time it takes to form cellular networks in our assay. Using an MTT or Trypan Blue proliferation assay, we found that the EVs^Z310^ have a small effect on NSC proliferation. Proliferation was increased by 10–15% within 24 h, depending on the source of the NSC and the concentration of EVs (Figure S4c,d). Adding EVs^MEF^ had no effect on NSC proliferation. Similar data were obtained with the Trypan Blue assay (Figure S4e). We conclude that the main effect of EVs is a cell shape change and cell network formation and, within the treatment time of <48 h, not a massive increase of cell proliferation.

### 3.3 Efficient dry‐drop assay to study the differentiation of NSCs

**FIGURE 6:**
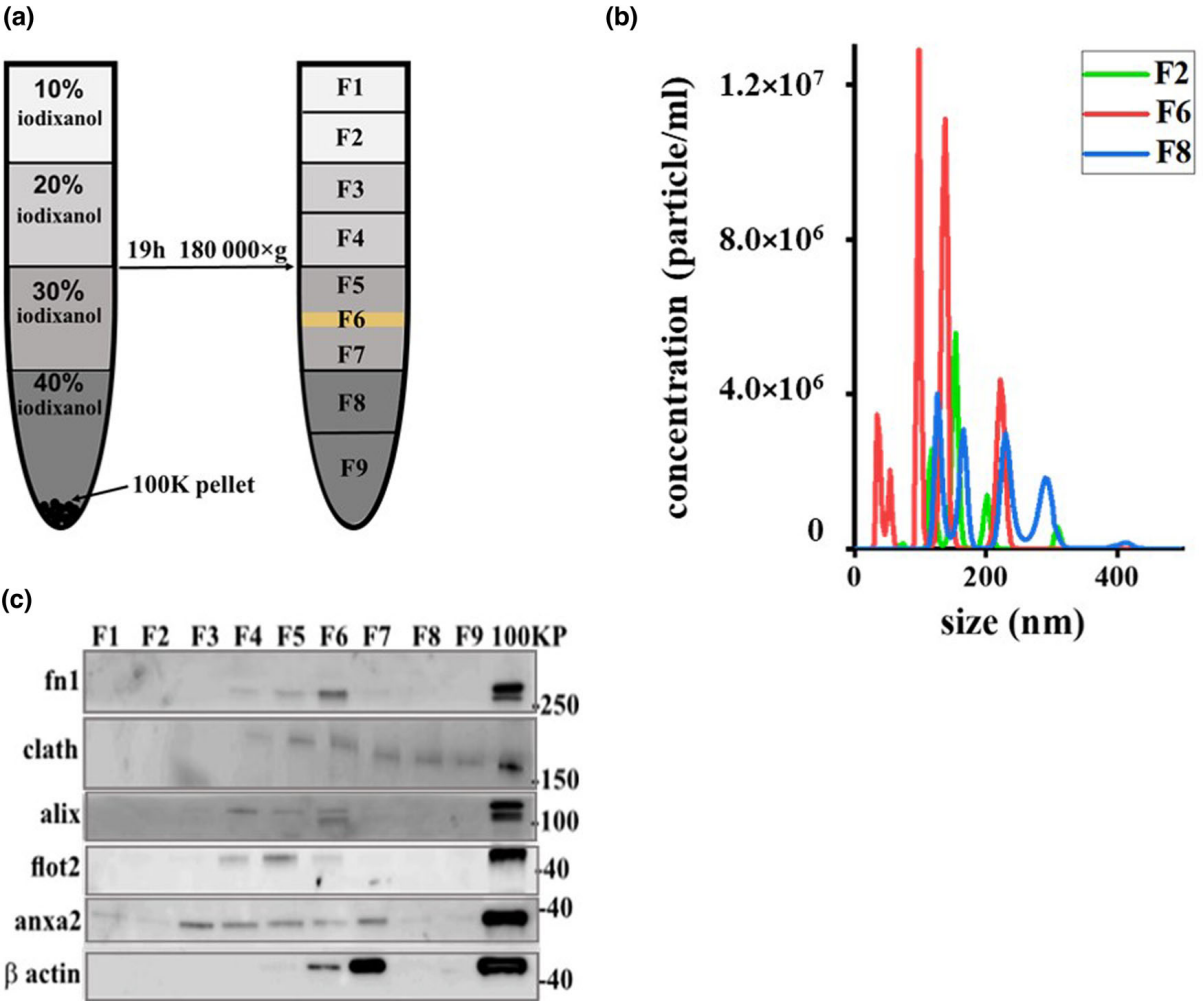
EV flotation on an iodixanol gradient produces sub‐types of EVs. (a) Scheme of EV^Z310^ purification by density gradient separation. The 100K pellet obtained by differential centrifugation was re‐suspended in 40% iodixanol and bottom loaded. After centrifugation, fraction 6 (yellow) was visible as an opaque band. (b) Particle size distribution in three fractions. Fraction 6 (red) showed the highest particle concentration and contained the majority of vesicles in the size range of 50–150 nm. Fractions 2 and 8 (green and blue) had a strong bias for larger vesicles (150–300 nm). (c) Western blot of the nine fractions and the starting material (100K pellet). Fraction 6 shows the highest level of fn1 and alix.

It is still considered challenging to purify EVs by centrifugation. A general recommendation is that EVs purified by differential centrifugation should be further purified on a density gradient, which separates vesicles according to their floatation speed and equilibrium density into vesicle sub‐populations and also removes contaminants (Kowal et al., 2016; Tkach et al., 2018). Therefore, the 100K EV^Z310^ pellet (Figure 2a) was suspended in iodixanol and subjected to iodixanol density gradient centrifugation (Figure 6a). After the centrifugation, the gradient was partitioned into nine fractions. Fraction 6 was an opaque band that could easily be seen and hence be recovered as a single fraction. In this fraction, nanoparticle tracking analysis revealed a high concentration of particles (red lines in Figure 6b) in a size range of 100–150 nm with smaller peaks at 50 and 220 nm. Fractions 2 and 8 contained mostly particles >150 nm. Thus, this method provides an enrichment of particles in the 50–150‐nm range. All nine fractions were analysed by Western blotting (Figure 6c). Clathrin was detected in fractions 4–9, flot2 in fractions 4–6 and both had maximum intensity in fraction 5. Fn1 and alix were detected only in fractions 4–6 and showed highest levels in fraction 6. Annexin2 was detected in fractions 3–7, and β actin in fractions 6 and 7. A comparison of the marker composition and particle size of the various fractions with the composition of the starting material showed that the iodixanol gradient further separated the EVs of the 100K pellet into biochemically diverse sub‐populations of EVs.

**FIGURE 7:**
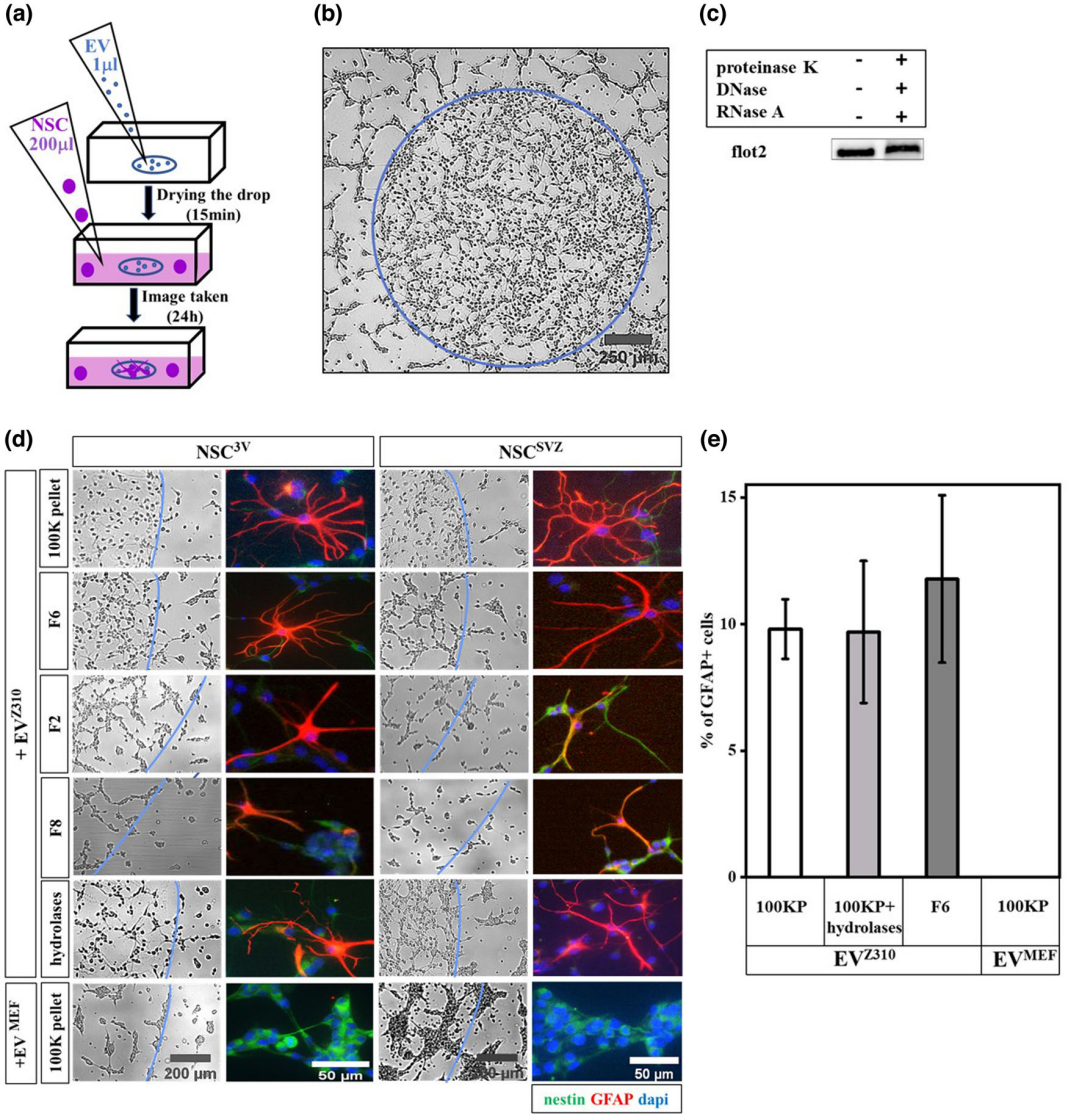
Dry‐drop assay to assess EV biological activity. (a) Dry‐drop assay: A 1 μL drop of EVs was deposited on the floor of an eight‐chamber slide and let dry for 15 min. Thereafter (for up to a week later if slides are kept at −20°C), 200 μL of the NSC suspension were added to each chamber. The chambers were incubated for 24 h, at 37°C in a tissue culture incubator. (b) The NSC^tz^ attached and formed a cellular network only in the area of the EV drop (blue boundary). Cells outside the boundary were still floating or were at best weakly attached. (c) Level of Flot2 located inside EVs^Z310^ was not affected by sequential treatment with proteinase K, DNase and RNase. (d) Fraction number 6 obtained from the iodixanol gradient provided excellent support for NSC^tz^ and NSC^SVZ^ network formation. When EVs^Z310^ from fraction 6 were used, immunofluorescence revealed the presence of cells with astrocyte morphology that also expressed GFAP (row 2). Sequential treatment with hydrolases had no influence on the ability of EV^Z310^ to evoke differentiation (row 5). EVs^MEF^ did not provide proper support for network formation, regardless of the type of NSC used (bottom row). The blue line marks the periphery of the EV^Z310^ dried drop. (e) Percentage of GFAP‐expressing cells in the area of the drop. Data were normalized to the cell number. Note the treatment with proteinase K and nucleases had no effect on the percentage of GFAP‐expressing cells.

To efficiently examine multiple EV fractions for their ability to induce differentiation of NSC^tz^ and NSC^SVZ^ cultures, we developed a material saving ‘dry‐drop’ activity assay. A 1‐μL drop of each EV fraction (protein concentration 76 μg/mL) was applied to the bottom of each chamber of an eight‐chamber tissue culture glass slide and left to dry for 15 min. Then, 200 μL of the NSC suspension (200,000 cells per millilitre) were added to the chambers and the slides were incubated for 24 h in a tissue culture incubator (Figure 7a). The organized network of attached NSCs^tz^ or NSCs^SVZ^ was readily visible and did not extend beyond the peripheral boundary of the dried drop (Figure 7b,d). The EVs from the 100K pellet and from fraction 6 provided excellent support for NSC attachment, network formation and robustly showed many attached cells that were GFAP‐positive (top two rows in Figures 7d and 7e first and third bar). This agreement means that EVs from the 100K pellet obtained by differential centrifugation and those in fraction 6 of the density gradient have a very similar NSC differentiation capacity, but fraction 6 of the iodixanol gradient was biochemically purer than the initial 100K pellet (Figure 6c). This additional purification will help in the eventual purification of the active factors. Note that NSC attachment, network formation and development of the GFAP‐positive astrocytes were less distinct for fractions 2 and 8 (Figure 7d, rows 3 and 4). The rest of the fractions were such that they lead to cell attachment only very sparsely and therefore only a few NSCs differentiated to astrocytes.

We also used the dry‐drop assay to determine, whether the differentiation activity is merely co‐purifying with EVs or is intrinsic to the vesicle. 100KP EVs were treated with proteinase and nucleases as previously described (Materials and Methods) (Shurtleff et al., 2017). A Western blot showed that Flot2 was proteinase K resistant, as expected, because this protein is contained in the interior of EVs (Figure 7c). Figures 7d (row 5) and 7e show that adding proteinase and nucleases to the EVs, had little effects on the percentage of the GFAP‐positive cells developing within the drop. These results indicate that EVs themselves and not a spurious contamination in 100K pellet is causal for the effect of EVs on NSC network formation and differentiation. We also examined EVs^MEF^ in the dry‐drop assay (Figure 7d, bottom row, and Figure 7e). Consistent with the results shown above (Figures 4a and 5a,b), EVs^MEF^ from the 100K pellet had no detectable activity in the dry‐drop assay. When slide chambers containing EV^Z310^ drops were stored at −20°C for a week, there was no reduction in NSC attachment, network formation and differentiation to GFAP‐positive astrocytes. Thus, the dry‐drop assay provides a convenient method that allows an economic use of EV preparations.

## 4 DISCUSSION

The interconnected, CSF‐filled cavities of the ventricular system (Figure 1) with their motile cilia‐powered transport machinery (Del Bigio, 2010) allow a targeted distribution of a variety of substances inside the brain. Many substances found in the CSF originate from the secretory epithelium of the choroid plexi that reach into the ventricular cavities. This secretome comprises metabolites, hormones, proteins, EVs, and so forth. Here we focus on two likely EV targets, the NSC niche of the SVZ (Doetsch et al., 1999; Obernier & Alvarez‐Buylla, 2019) and the NSCs that reside in the tanycyte region (Haan et al., 2013; Robins et al., 2013). Both niches are in contact with CSF and hence in contact with EVs. We established an assay in which EVs^Z310^ secreted by the Z310‐choroid plexus cell line were brought into contact with NSCs obtained from neurospheres that were generated either from the SVZ or the tanycyte region. Both, NSC^SVZ^ and NSC^tz^ formed, within 24 h, complex cellular networks that begun to express neuronal (Tuj1, *Cdh2*) and astrocytic markers (GFAP, *Gfap*). The differentiation‐inducing activity resided in EV^Z310^ with a diameter of 50–150 nm that was purified by differential centrifugation and/or iodixanol density gradient centrifugation. The activity contained in EV^Z310^ was resistant to DNase, RNase and proteinase K treatment, suggesting that the inducing activity was an EV component protected by the EV bilayer membrane and was not a contaminant co‐purifying with the EVs. It should be recalled that the NSC^SVZ^ used in our study are a mixture of quiescent, active NSCs and transit‐amplifying cells that differ in both, expression levels of marker genes and the rate of proliferation (Silva‐Vargas et al., 2016). In the case of NSC^tz^, there is presently no evidence for the existence of such sub‐types. Our neurospheres may not fully replicate a NSC niche because they are deprived of the influence of other niche cells (Langlet et al., 2013; Obernier & Alvarez‐Buylla, 2019). Nevertheless, neurospheres have been effectively used in many studies investigating the process of NSC differentiation (Haan et al., 2013; Lepko et al., 2019; Robins et al., 2013; Silva‐Vargas et al., 2016).

Our use of a choroid plexus cell line and of primary cultures has some limitations. Choroid plexus is the main producer of CSF but not the only one (Damkier et al., 2010). CSF‐born EVs also originate, for example, from ependymal cells that form the walls of the ventricles. Such EVs are obviously missing in our preparation. Z310 cells are used as a surrogate for choroid plexus and they are derived from adult choroid plexus tissue (see references in Materials and Methods). In the adult animal, the composition of CSF, and by implication the cargo of CSF‐born EVs will depend on physiological state of the organism (Cravatt et al., 1995; Myung et al., 2018; Tietje et al., 2014; Zhang et al., 2017). It is unlikely that the Z310‐derived EVs reflect this complexity in full. Nevertheless, we were able to isolate a biological activity (EV^Z310^ and EV^CHP^) that rapidly induces cell networks from two types of NSCs. EV^CHP^ was as effective in this process as EV^Z310^, so the Z310 cells can be used as substitute source for choroid plexus‐born EVs. This activity purifies over multiple centrifugation steps and yields a distinct EV fraction containing vesicles in 50–150‐nm range. This simple purification scheme can readily be extended to a whole spectrum of other types of EVs isolated from different cells (astrocytes, ependymal cells) and from other brain tissues. Our high‐throughput dry drop assay offers a test bed for cellular responses such as the formation of cellular processes or the expression of particular marker genes.

EVs^MEF^ secreted by MEFs had only a minor effect on NSCs. Cells formed small aggregates but never any of those cellular networks seen after treatment with EVs^Z310^ and EVs^CHP^. In addition, none of the neural/glial differentiation markers was induced by EVs^MEF^. EVs^Z310^ and EVs^MEF^ showed marked differences in their protein composition. This difference might shed light on the factors that evoke cell differentiation. The most significant difference was the presence of ∼250 ‘membrane’ proteins in the EV^Z310^. String analysis of possible protein–protein interaction revealed interacting proteins involved in ‘neurogenesis’, ‘cell differentiation’, ’membrane trafficking and organization’.

Slit homolog 2 protein (Slit2), which is one of the proteins identified only in EV^Z310^ cargo by mass spectrometry and Western blotting, could contribute to the formation of cell processes that characterize the cell network. Slit proteins serve as repulsive axon guiding molecules via Slit‐Robo signalling and it was suggested that choroid plexus is a source of Slit (Kaneko et al., 2018; Nguyen‐Ba‐Charvet & Chédotal, 2002; Sawamoto et al., 2006). Synaptogenesis is required for proper neuron function and we identified several synaptic proteins in EV^Z310^, such as synaptotagmin1, and 2, and the synaptic vesicle membrane protein VAT‐1. Synaptotagmins are calcium sensors participating in triggering neurotransmitter release at the synapse and also play a role in synaptic vesicle trafficking.VAT‐1 is involved in synaptic vesicle transport.

EVs^Z310^ induced astrocytes that expressed a characteristic marker, GFAP. *Gfap* transcription is up‐regulated by the transcription factor Stat3 and the transcriptional activator Brg1 (also known as Smarca4) (Brenner & Messing, 2021; Ito et al., 2018). Stat3 and Smarca4 were identified by MS in EV^Z310^ and confirmed by Western blotting. Stat3 also regulates *cdh2* expression via Jak/Stat pathway (Loh et al., 2019) and our qPCR data showed that *cdh2* expression was up‐regulated upon EV^Z310^ addition. The EV^Z310^ also caries integrins and small GTPases, such as Ras Homolog Gene Family Member A (Rhoa) and RAC, which also regulate *cdh2* expression (Barcelona‐Estaje et al., 2021). *Vimentin* is also induced by Stat3 signalling (Wu et al., 2004). While GFAP and Cdh2 were not identified in EV^Z310^, these vesicles contain vimentin. There is also vimentin in NSCs prior to their exposure to EV^Z310^. In this case, vimentin seen after EV‐treatment could be a mixture of cellular and EV‐derived vimentin. In our assays, we observed intense fn1 staining at or near the tip of the cellular processes. Such staining may arise from fibronectin1 contained in EV^Z310^ that locally aggregate. Note, however, that cells also express and secrete endogenous fn1 that could also be present at the tip, either inside or outside of the cell. Other adhesion proteins and scaffolding proteins (integrins, tetraspanins) are present in EVs^Z310^ and could act as guidance cues that control the outgrowth of the nascent NSC processes. Growing tips of axons can rapidly detect and react to the local guidance cues (Holt et al., 2019). Such guidance cues can also initiate local synthesis of different proteins in the distal part of axons (Cagnetta et al., 2018). In this case, EVs may not be taken up by the NSCs, but are acting as exogenous guidance posts. There are precedents for cellular responses to EVs that do not require EV internalization (Margolis & Sadovsky, 2019; Mckelvey et al., 2015). EVs may collapse on the cell surface and release the protein cargo that subsequently interacts with cell surface receptors (Alabi & Tsien, 2013).

Since the purified EVs are heterogeneous, only a sub‐set of the EV^Z310^‐specific proteins would be present in any given EV. EVs with a diameter of 150 nm have a volume that is nearly fifty times greater than that of a synaptic vesicle (40 nm) that contains about 160 protein molecules (Takamori et al., 2006; Taoufiq et al., 2020). Hence, a single EV could contain thousands of protein molecules. Assuming that the NSC targets incorporate multiple EVs, collectively such vesicles could confer entire signalling pathways to the target cells. In developmental biology, and the change in cell morphology is a developmental process, multiple signals converge on a single tissue. For example, the developing limb of vertebrates is controlled by a combination of growth‐ and transcription factors (Zuniga & Zeller, 2020). In this case, the signals are not packaged in EVs but are soluble local mediators. This is different for the NSC niches in the adult brain where signals must travel several millimetres even in mice and much farther in larger brains. A long‐range transport of the multiple signals would benefit from an enclosure of signals in a carrier vesicle.

Induction of *gfap, stat3* and *fn1* expression was dependent on the amount of EVs added to the culture. Such dose dependence and the accompanying saturation suggest that EVs are involved in a receptor‐mediated process. This could be an interaction with cell surface receptors or receptor‐mediated EV uptake (Russell et al., 2019). Work by others shows that target cells internalize EVs in a time‐ and dosage‐dependent manner (Bonsergent et al., 2021; Song et al., 2021). Bonsergent et al. suggested that EV uptake is a low‐yield process, and only about 30% of internalized EVs were capable of delivering their contents. In their study, a dose response was observed but saturation could not be reached, even when a dose >100 μg/mL (doses refer to EV protein content) was used (Bonsergent et al., 2021). Song et al. observed that EV uptake by mouse embryonic stem cells was dose‐ and time‐dependent over a 1‐day period. EV internalization was detected at an EV level of 2 × 10^9^ but increased significantly when the concentration was fivefold higher (Song et al., 2021). Sharma et al. showed that EV addition rescued the decrease of the total cell number and neurons in a loss‐of‐function neural model in dose‐dependent manner (47.5–190 μg/mL) (Sharma et al., 2019). EV^MEF^ were inactive in our assay, even when a very high dose (608 μg/mL) was provided. The cause for this may be that the NSC target and fibroblast‐derived EVs^MEF^ are not compatible. The combination of choroid plexus‐derived EVs^Z310^ and NSCs, however, reproduces a naturally occurring situation. At a dose of 76 μg/mL protein (approximate the ED_50_), a dry drop contains 1.6 × 10^6^ EVs. A typical dry drop contains 2500 NSCs. Thus, there are ∼800 EVs per cell. As pointed out above, EVs are heterogeneous and only a fraction may be acting in the network forming process. Thus, a rather small number of EVs may be sufficient to evoke cell differentiation.

Adding EVs^Z310^ suspended in buffer to the NSCs or, alternatively providing EVs as a dried‐down drop‐induced network formation. Even when the dry drops are kept at −20°C for a week, they were still very active. This indicates that the activity is stable in the EV for a significant length of time. Thus, our dry‐drop assay allows testing of diverse EVs and multiple responsive cells in a short time and with minimal amounts of testing material. Moreover, this assay is high throughput. This allows a more efficient purification of EV proteins and other factors (e.g., miRNAs (Lepko et al., 2019; Yagi et al., 2017)) that mediate the effects that EVs exert on their target cells.

## AUTHOR CONTRIBUTIONS

Zuzana Ditte and Gregor Eichele designed the study. Zuzana Ditte performed most of the experiment. Ivan Silbern performed mass spectrometric analysis. Zuzana Ditte and Peter Ditte performed qPCR. Peter Ditte isolated MEF. Zuzana Ditte, Ivan Silbern, Peter Ditte, Henning Urlaub and Gregor Eichele analysed and interpreted data, and revised the content critically. Zuzana Ditte and Gregor Eichele wrote the manuscript with inputs from other authors. All authors read and approved the final version of the manuscript.

## CONFLICT OF INTEREST

The authors declare that there is no conflict of interest.

## Supporting information

Supporting Information

Supporting Information

## Data Availability

The mass spectrometry data have been deposited on the ProteomeXchange Consortium via the PRIDE (Perez‐Riverol et al., 2019) partner repository with the dataset identifier PXD031862.
