# Supplementary material for: Extracellular vesicles derived from the choroid plexus trigger the differentiation of neural stem cells
Source: J Extracell Vesicles. 2022 Nov 2;11(11):12276. doi: 10.1002/jev2.12276 (PMC9630752; doi:10.1002/jev2.12276)

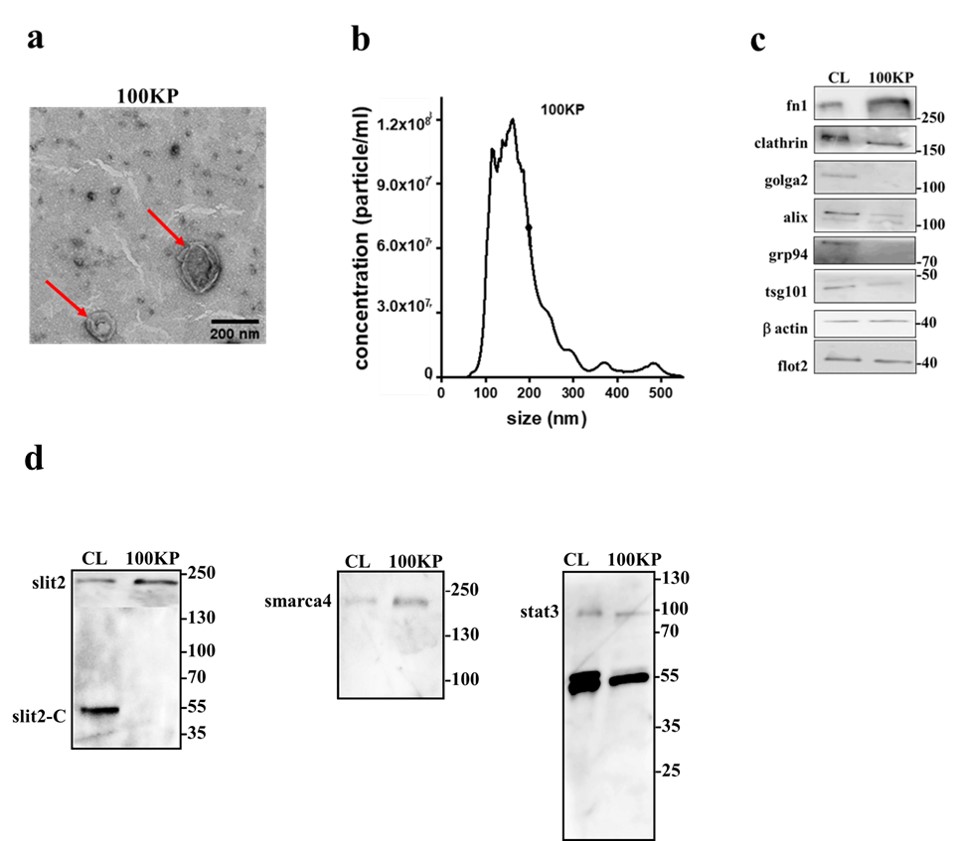


**Supplementary Figure 1. Purification and characterization of EV^MEF^ from MEF cell conditioned medium.**

(a) Representative electron micrograph of vesicles (red arrows) present in EV^MEF^ 100K pellet.

(b) Nanoparticle tracking analysis data showing the size distribution of the particles in EV^MEF^ 100K pellet. The 100K pellet obtained after ultracentrifugation, shows EVs in a size range of 75nm-200nm.

(c) 100K pellet obtained after differential centrifugation was analyzed by Western blotting together with MEF cell lysate (CL) for EV positive and negative markers.

(d) Western blot of the Z310 cell lysate (CL) and EVs^Z310^ in the 100K pellet (100KP in Figure 2(a)) showed that selected proteins give specific bands at molecular weights provided by the manufacturer of the antibodies. Slit 2: 200kDa, smarca4: 185kDa and stat3 92kDa. Note, the specific bands are present in the cell lysate and the EVs^Z310^.


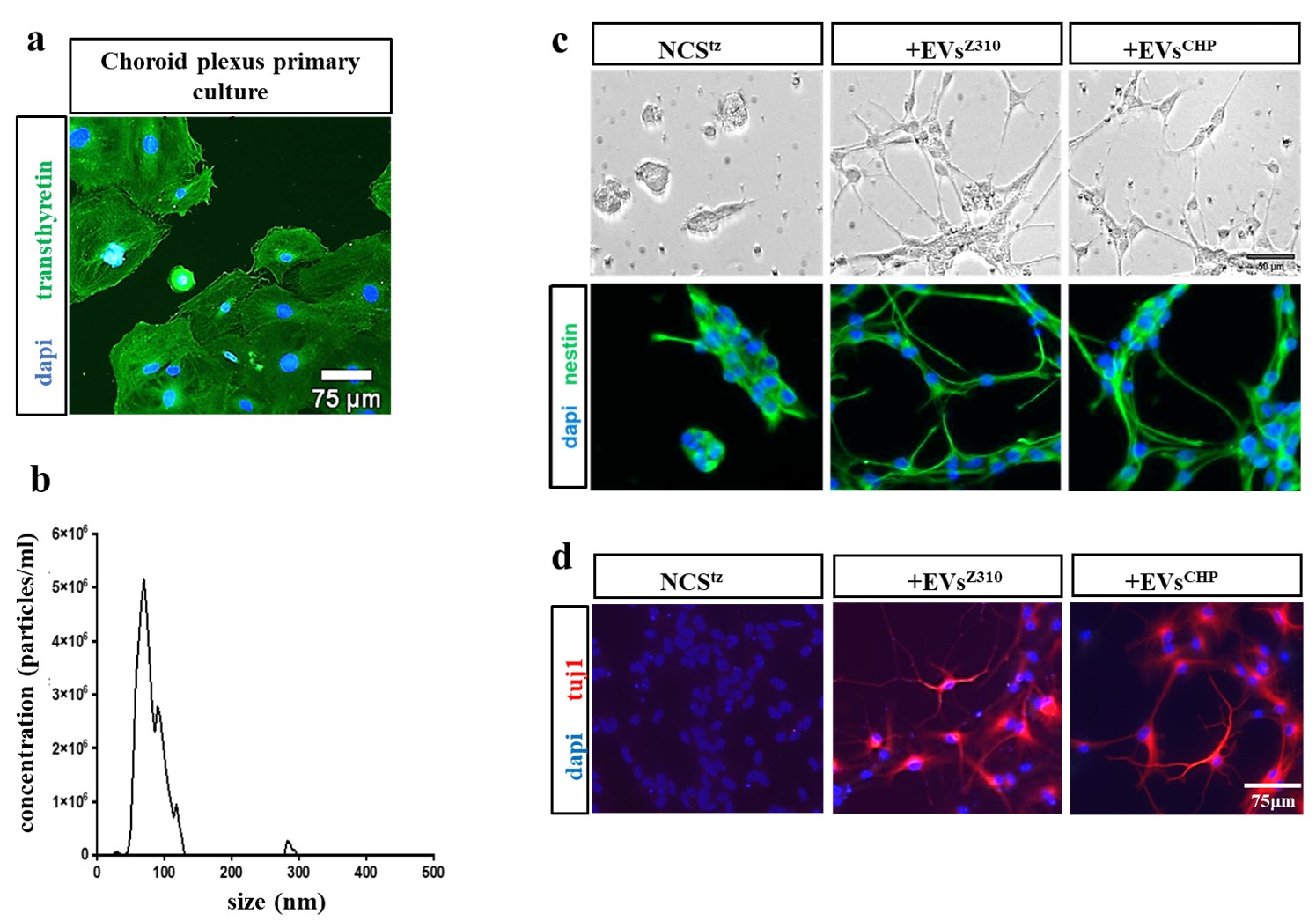


**Supplementary Figure 2: EVs produced by primary culture of choroid plexus (EVs^CHP^) induce NSC differentiation.**

(a) Immunostaining for the choroid plexus marker transthyretin (green) in all primary culture cells.Nuclei: DAPI (blue)

(b) Nanoparticle tracking analysis showing the size distribution of EVs^CHP^ 100K pellet. The size ranges between 50-120nm.

(c) EVs^CHP^ induce NSC^tz^ differentiation reminiscent of that seen after treatment with EVs^Z310^  (compare the middle and right column). Note the formation of networks of NSC^tz^ cells. Top bright field, bottom nestin staining (24h after adding EV^CHP^).

(d) Both EVs^CHP^ and EVs^Z310^ induce tuj1 expression in NSC^tz^ (48h).

**
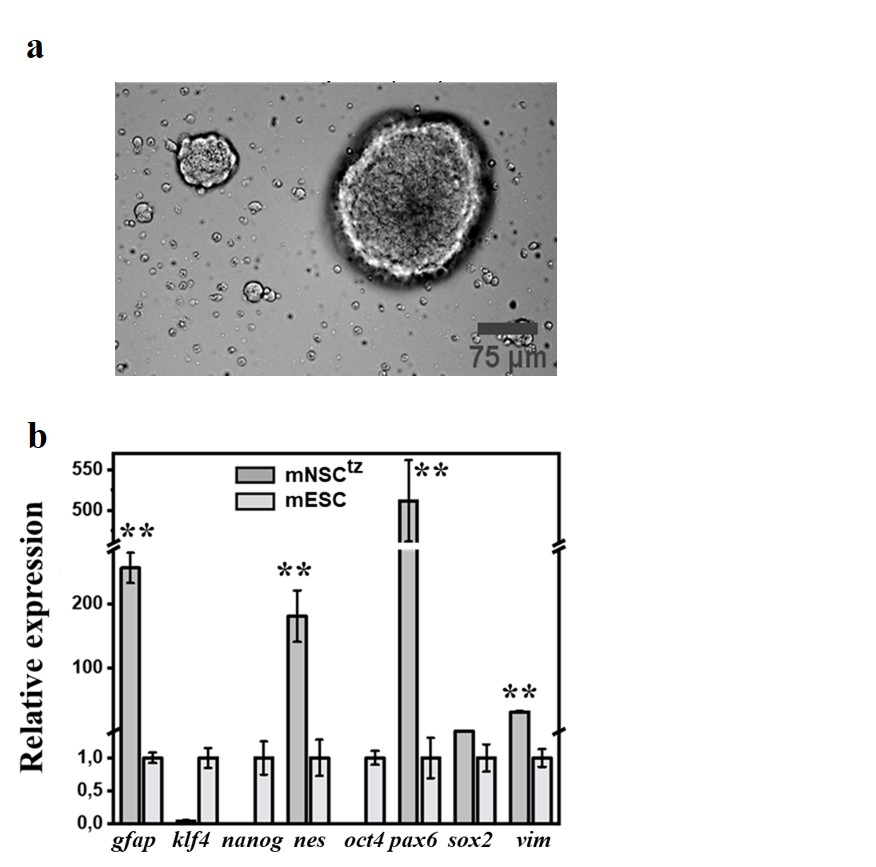
**

**Supplementary Figure 3: Murine NSC^tz^ form neurospheres and express neural stem cell markers.**

(a) Bright field image of the spherical NSC^tz^ aggregates (neurospheres) cultured in a low attachment surface dish.

(b) qPCR showed significant higher level of *Gfap, Pax6, nestin* and *vimentin* expression in NSC^tz^ when compared to embryonic stem cells (ESC). Values are presented as means ± SD, three biological and three technical replicates were analyzed (** p< 0.01, Student´s t test).


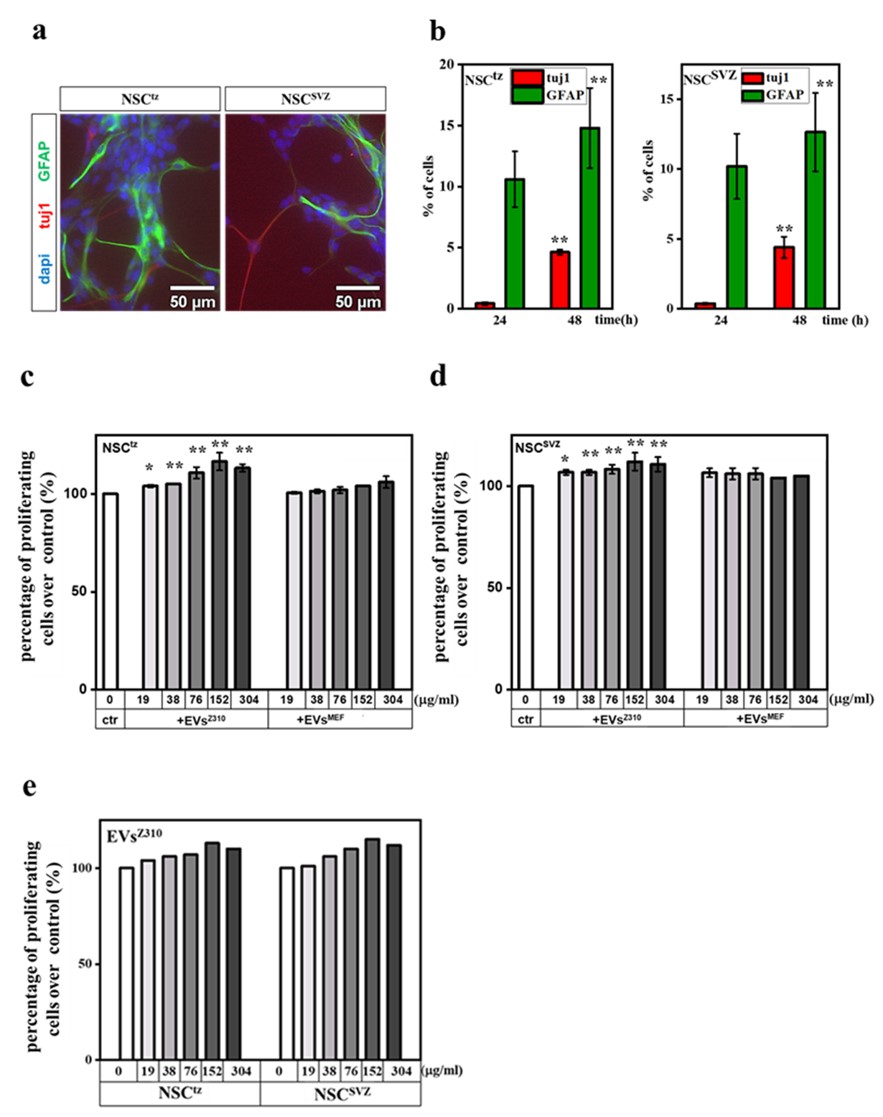


**Supplementary Figure 4. Effect of EV treatment of NSC^tz^ and NSC^SVZ^ on the expression of Tuj1 and GFAP (a, b) and on the rate of cell proliferation (c-e).**

(a) Examples of NSC^tz^ and NSC^SVZ^ cultures in the presence of EVs^Z310^ (total protein concentration was 304μg/ml) and immunostained for Tuj1 (neurons) and GFAP (astrocytes) after 48h of treatment.

(b) Quantification of percentage of GFAP+ cells (astrocytes) and Tuj1+cells (early neurons) in NSC^tz^ and NSC^SVZ^ after co-culture with EVs^Z310^ (total protein concentration 304μg/ml) for 24 and 48h. Values are presented as means ± SD (** p< 0.01, Student´s t test).

(c) MTT proliferation assay showed that after 24h of treatment, EVs^Z310^ significantly induced NSC^tz^ proliferation. EVs^MEF^ did not induced NSC^tz^ proliferation above the control levels. Values are means ± SD, 4 biological and 4 technical replicates were analyzed (* p < 0.05, ** p< 0.01, Student´s t test).

(d) MTT proliferation assay showed that after 24h of treatment EVs^Z310^ but not EVs^MEF^ significantly induced NSC^SVZ^ proliferation. Values are presented as means ± SD, n=16 (* p < 0.05, ** p< 0.01, Student´s t test).

(e)Trypan blue proliferation assay showed that EVs^Z310^ induce NSC^tz^ and NSC^SVZ^ proliferation after 24h.

**Supplementary information**

**Material**


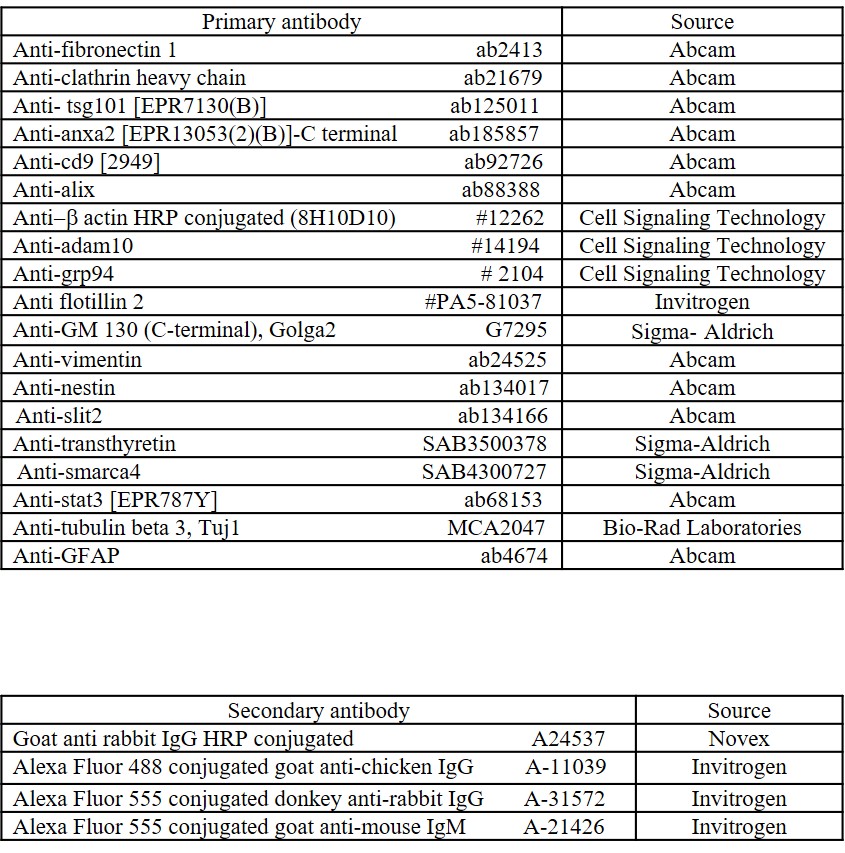


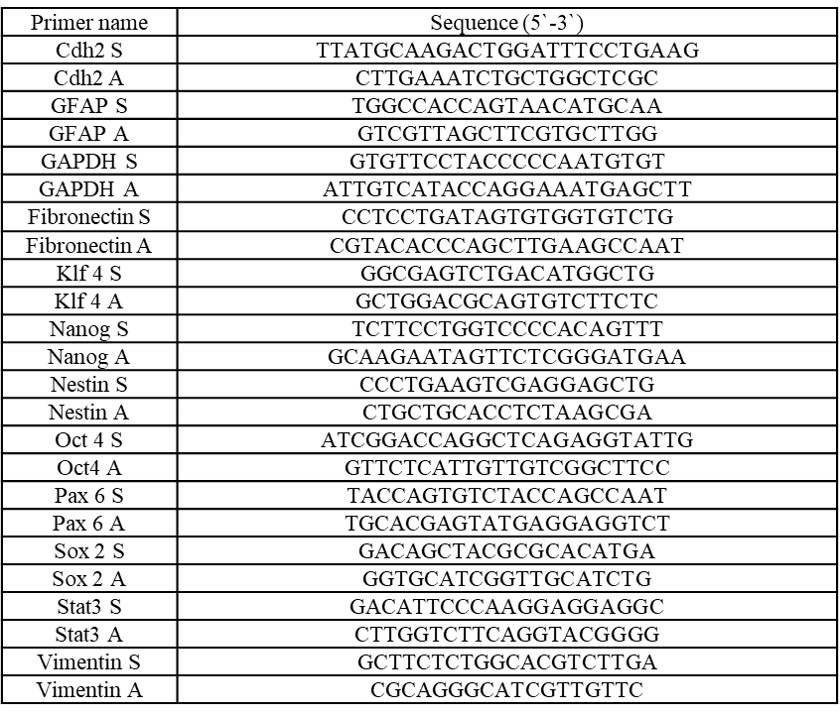

Supplement: Supplementary file 1 — Supporting Information [file JEV2-11-12276-s001.docx]
